# Secretome analysis of chickpea reveals dynamic extracellular remodeling and identifies a Bet v1-like protein, CaRRP1 that participates in stress response

**DOI:** 10.1038/srep18427

**Published:** 2015-12-18

**Authors:** Sonika Gupta, Vijay Wardhan, Amit Kumar, Divya Rathi, Aarti Pandey, Subhra Chakraborty, Niranjan Chakraborty

**Affiliations:** 1 National Institute of Plant Genome Research, Aruna Asaf Ali Marg, New Delhi-110067, India.

## Abstract

Secreted proteins maintain cell structure and biogenesis besides acting in signaling events crucial for cellular homeostasis during stress adaptation. To understand the underlying mechanism of stress-responsive secretion, the dehydration-responsive secretome was developed from suspension-cultured cells of chickpea. Cell viability of the suspension culture remained unaltered until 96 h, which gradually declined at later stages of dehydration. Proteomic analysis led to the identification of 215 differentially regulated proteins, involved in a variety of cellular functions that include metabolism, cell defence, and signal transduction suggesting their concerted role in stress adaptation. One-third of the secreted proteins were devoid of *N*-terminal secretion signals suggesting a non-classical secretory route. Screening of the secretome identified a leaderless Bet v 1-like protein, designated CaRRP1, the export of which was inhibited by brefeldin A. We investigated the gene structure and genomic organization and demonstrated that CaRRP1 may be involved in stress response. Its expression was positively associated with abiotic and biotic stresses. CaRRP1 could complement the aberrant growth phenotype of yeast mutant, deficient in vesicular transport, indicating a partial overlap of protein secretion and stress response. Our study provides the most comprehensive analysis of dehydration-responsive secretome and the complex metabolic network operating in plant extracellular space.

Secreted proteins are considered to be indispensable for multitude of biological and physiological processes, functioning in signal transduction, and understanding their language may uncover various signaling networks^1^. The secretome constitutes an important class of bioactive molecules, proteins in particular, that regulates growth, development, cell division and differentiation besides defense and stress related responses, among others. The secreted proteins are believed to function both locally and systemically^2^. Despite the important biological role employed by the secreted proteins, the characterization of stress-responsive secretome has so far received little attention. Cell secretion allows the release of intracellular proteins and metabolites, into the extracellular space (ECS), presumably to sense and respond to the changing environment^3^,^4^. Increasing evidence suggests that there is continuous cross-talk between ECS and the cytoskeleton network^5^. The signals in the ECS not only function during the course of normal cellular development but they can act in response to different environmental stimuli. The suppression and/or accumulation of such stress-responsive proteins may lead to discovery of potential biomarkers for stress progression/adaptation. Biomarker discovery for human diseases^6^,^7^ and plant pathogenesis^8^ are important instances.

Plants, as sessile organisms, are endowed with highly sophisticated biological mechanisms to precisely regulate their growth and development in response to environmental risk factors. Among several abiotic stresses, water-deficit or dehydration is the fundamental factor that negatively affects plant productivity resulting in major yield loss of foremost crops worldwide^9^,^10^. Dehydration response in plants is a complex phenomenon, and the exact structural and functional modifications caused by dehydration are poorly understood. Plants render dehydration tolerance either by escaping or by maintaining a favourable internal water balance^11^,^12^. The changes at molecular level are regulated by a number of different, and potentially overlapping, signal transduction pathways^13^,^14^. Interestingly, dehydration induces the expression of proteins not specifically related to this stress, but rather to reactions against cell damage^15^. The dehydration-responsive cellular cross-talk is thought to be modulated by a diverse population of secreted proteins, and thus, expression profiling of proteins has become an important tool to investigate various stress-responsive signaling networks.

The analysis of secretome appears to be a fundamental approach to map the quality and quantity of the extracellular proteins, which provides insight into possible biological pathways involved. The secretomes of plants submitted to stress condition usually contain significantly more leaderless secretory proteins (LSPs) than the secretomes of unstressed plants^3^. During the past few years, there have been rapid advances in plant secretomics^16^,^17^,^18^. While most research interest in the secreted proteins has been restricted to the biotic stress conditions, the study of the effect of abiotic stress remained highly limited. Interestingly, recent studies of plant proteomics have enhanced our understanding of the molecular basis of abiotic stress response^14^,^19^.

Developmental control of protein expression in differentiated tissues is assumed to mask the identity of proteins that are differentially expressed as part of stress response. Therefore, investigation of stress-responsive proteins in undifferentiated cells, particularly in suspension-cultured cells (SCCs) has potential for: (i) identifying novel proteins related to stress which have not yet been characterized and (ii) recognizing already characterized proteins that have not been identified as stress-responsive proteins. In a previous study, we developed the secretome of chickpea to propose basic model for plant processes and expression system for recombinant proteins of commercial importance^20^. The present study was aimed at identifying dehydration-induced secreted proteins in the chickpea SCCs and novel components involved in such stress tolerance. Using a combinatorial approach of 1- and 2-DE techniques coupled with LC-MS/MS, we identified 215 dehydration-responsive proteins that comprise expected and unexpected secretory proteins. Screening of the secretome led us to a Bet vI-like ripening related protein, designated CaRRP1. We investigated the gene structure and organization of CaRRP1 and demonstrate, for the first time, that it might play key role in intracellular stress adaptation. The expression analysis revealed that CaRRP1 is involved in multivariate stress response, which might help in the long-term efforts to develop transgenic crops with improved stress tolerance.

## Results and Discussion

### Imposition of water-deficit condition in SCCs

There has been much interest in the ability of eukaryotic cells to regulate their internal water potentials^21^. Although considerable knowledge of this process in certain algae is available^22^, our understanding of dehydration regulation in higher plants is much more limited. While suspension cultures may be considered as over simplification of the complex mechanisms involved in dehydration response, they represent a highly controllable and homogeneous experimental system. However, there is little information on the degree of dehydration and physio-biochemical characteristics of suspension culture. Sugar alcohols such as mannitol and sorbitol act as common source of carbon and energy and also, as osmotica in response to environmental stress including dehydration^23^. On the contrary, PEG is known to mimic dehydration by reducing water availability and modifying the osmotic potential of nutrient solution^24^. We therefore used PEG as an ideal osmoticum to model dehydration conditions in chickpea suspension culture.

**Figure 1:**
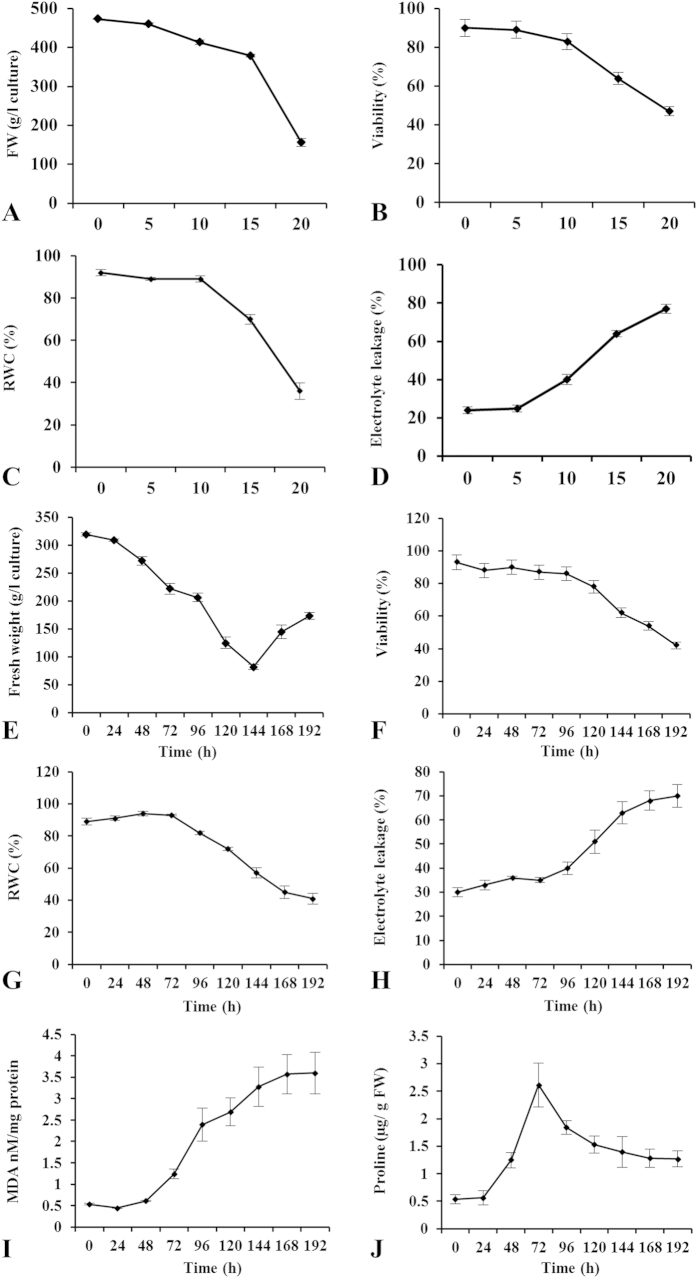
Physiochemical changes after exposure to PEG-induced dehydration in callus cultures. The SCCs were maintained at 25 ± 2 °C, at 16 h photoperiod and treated with varying concentrations of PEG. Quantitative determination of (**A**) callus fresh weight, (**B**) cell viability, (**C**) RWC and (**D**) electrolyte leakage was carried out with increased PEG concentration. Comparative estimation of (**E**) fresh weight, (**F**) cell viability, (**G**) RWC, (**H**) electrolyte leakage, (**I**) MDA and (**J**) proline content at 10% PEG. The experiments were accomplished in triplicates (n = 3) and average mean values were plotted. Vertical bars in the graphs indicate the S.E.

To quantitate the influence of dehydration on the physiology of SCCs, the culture was subjected to various degree of dehydration at varying PEG concentration (0 to 20%). The culture was initially examined for change in fresh weight. No obvious deleterious changes could be observed up to 10% PEG in the media. Subsequent increase in the PEG concentration resulted in sharp decrease in fresh weight (Fig. 1A). Furthermore, viability of the suspension culture demonstrated a positive correlation with the fresh weight measurements. Quantitative determination of cell death indicated that more than 80% cells were viable up to 10% PEG treatment (Fig. 1B). The relative water content (RWC) of the suspension culture was 92% in unstressed condition, which marginally decreased at 10% PEG. However, RWC was drastically decreased to 70% and 36% at 15% and 20% PEG treatments, respectively (Fig. 1C). The electrolyte leakage was found to be maintained at an almost constant level until 5% PEG treatment and showed a marginal increase at 10% PEG. However, a steady increase was observed in 15% and 20% PEG treatments (Fig. 1D). These results indicate that the treatment of 10% PEG imparted moderate dehydration stress, but higher concentrations (15–20%) might cause severe damage (Fig. 1A–D).

To compute the temporal effect of dehydration on the physiology of culture, 10% PEG treated suspension culture was examined up to 192 h. The morphometric analysis did not show any visible change in the SCCs until 72 h of dehydration (Supplementary Fig. 1A). The culture was further analyzed microscopically for cellular integrity and homogeneity (Supplementary Fig. 1B). Approximately 90% of the cells were viable until 72 h of dehydration, when stained with fluorescein diacetate (FDA) and counterstained by Evan’s blue (Supplementary Fig. 1C,D). A time-dependent decline in the fresh weight was observed except for a sudden increase after 144 h (Fig. 1E). This increase might be due to the initiation of second growth cycle of the culture. Cell viability was found to be 86% until 96 h whereas the cell death increased promptly during 120–192 h (Fig. 1F). RWC was found to correlate closely with the growth measurements. There were no significant changes in the RWC during the first 48 h of dehydration. However, a marginal increase in RWC was observed around 48–72 h followed by a gradual decline during later stages (Fig. 1G). A constant level of electrolyte leakage was maintained up to 72 h, but it increased linearly with successive time points (Fig. 1H). Furthermore, lipid peroxidation in terms of MDA accumulations displayed maximum maintenance of cell membrane integrity up to 48 h, portraying a mild stress on the cultured cells. However, the damage aggravated at later stages of 96–120 h (Fig. 1I). Increased levels of compatible solutes after dehydration challenge are common in both intact plants and callus suspension culture^25^,^26^. Therefore, proline accumulation in the suspension culture was determined across different time points (Fig. 1J). Upon dehydration, proline accumulation curve displayed a peak at 72 h but dropped afterwards. The increase in proline accumulation might cause probable osmotic adjustment, which was also reflected in the increase in RWC during that period.

**Figure 2:**
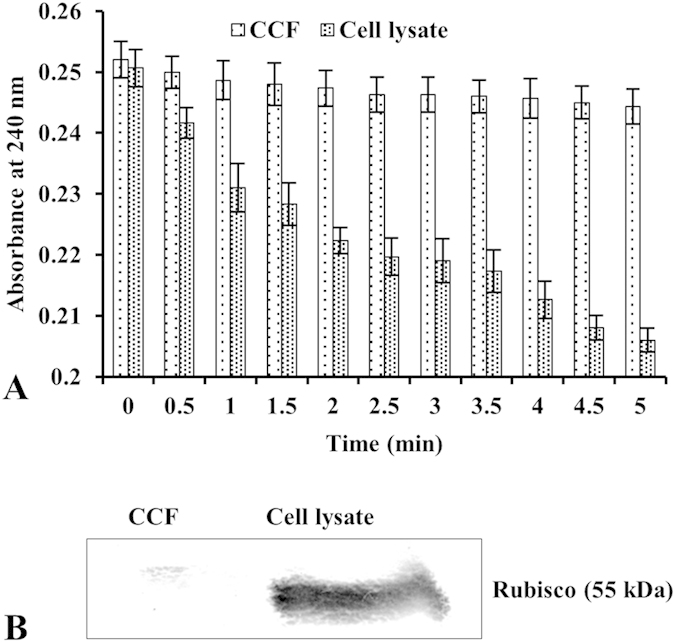
Purity evaluation of chickpea secreted fraction. (**A**) Determination of catalase-specific activity in cell lysate and secreted fractions. The SCCs were collected after centrifugation at 3,000 g for 10 min at 4 °C and cell lysates were used as positive control. (**B**) Immunoblot analysis of secreted proteins with anti-RbcL antibody. An aliquot of 50 μg protein was separated by 12.5% SDS-PAGE and electroblotted onto nitrocellulose membrane. Rubisco was detected using alkaline phosphatase conjugated secondary antibody. Vertical bars in the graphs indicate the S.E.

The purity of the secretome was examined by catalase assay as an intracellular marker. The secreted proteins from dehydration treated suspension culture did not show any significant catalase activity, while proteins prepared from the calli showed high activity (Fig. 2A). We also examined presence of Rubisco, a nonsecretory protein, which could possibly contaminate the secreted proteins. Protein samples were separated by SDS-PAGE and probed with anti-Rubisco antibody. Rubisco was detected in the proteins extracted from calli but not in the secreted fraction (Fig. 2B), indicating that the fraction was essentially free from intracellular proteins.

### Identification of dehydration-responsive secreted proteins

It has been frequently observed that dehydration causes simultaneous activation of specific protein synthesis and inhibition of the synthesis of some constitutive cellular proteins^27^. The expression of dehydration-responsive proteins in the suspension culture was thus monitored using 1- and 2-DE analyses. We attempted to resolve the proteins initially onto a basic pH range (pH 6–11) (Supplementary Fig. 2), which exhibited poor resolution. Henceforth, the proteins were separated onto a pH range of 4–7 (Supplementary Fig. 3). While 110 spots were detected in unstressed condition, 104 spots could be classified as of high quality (Supplementary Table 1). After an automated spot detection, the protein spots were checked manually in order to eliminate any possible artifacts such as gel background or streaks. The spot densities were normalized against the total density present in the respective gel to overcome the experimental errors. To make comparison between the stressed and unstressed samples, a second level matchset was created (Supplementary Fig. 4). The intensity of spots was normalized to that of landmark proteins used for internal standardization. The filtered spot quantities from the higher level matchset were assembled into a data matrix that consisted of 106 spots indicating change in intensity for each spot. The data revealed that nearly 95% of the spots on the reference gels were of high quality, reflecting the reproducibility of the experimental replicates (Supplementary Table 1). A total of 100 protein spots were reproducibly detected across unstressed and dehydration conditions. Quantitative image analysis revealed a total of 24 protein spots that changed their intensities significantly by more than 2.5-fold. While most spots showed quantitative changes, several spots also showed qualitative changes. Most of these differential spots were low abundant, out of which 8 spots could be processed for downstream analysis. The MS/MS analysis could identify 13 proteins, which are listed in Supplementary Table 2. The secreted proteins were also resolved on 12.5% SDS-PAGE, and gel slices were subjected to MS/MS analysis. Using 1-DE approach, 202 proteins were identified, which would have been largely underrepresented utilizing 2-DE based separation alone (Supplementary Table 3).

### Physicochemical properties of the dehydration-responsive proteins

**Figure 3:**
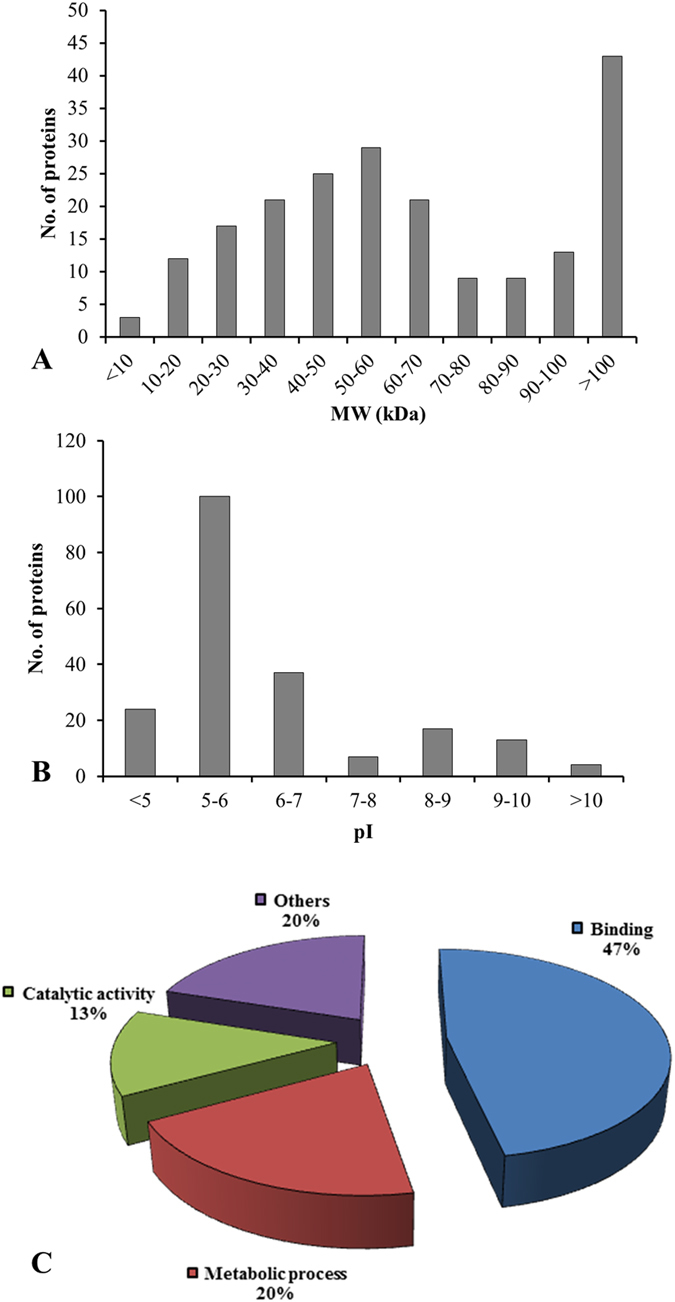
Characteristic features of secreted proteins and their functional classification. Ranges of molecular weight (**A**) and pI (**B**) of proteins identified in the secretome. Most of the proteins identified have pI between 5 and 10 and a wide range of molecular weight distribution. The identified proteins were assigned a putative function using Pfam and InterPro databases and functionally categorized as represented in the pie chart (**C**). Percentage of proteins in each assigned class was calculated based on the number of assigned proteins over the total proteins.

The 202 dehydration-responsive secreted proteins varied in molecular weight with significant increase in the secretion of high molecular weight proteins. In stressed as well as unstressed conditions, maximum number of the identified secreted proteins has relatively high molecular weights in the range >100 kDa (Fig. 3A). However, the isoelectric points (pIs) of the proteins ranged from 4 to 12 with maximum proteins in pI range 5–6 (Fig. 3B). The secretion of acidic proteins was found to be induced by dehydration that accounted for 124 proteins in the pI range of 4 to 6. Many secreted proteins had a pI of more than 8, which corroborate the findings of previous report^28^.

### Localization prediction of dehydration-responsive secreted proteins

Differentially regulated secreted proteins were examined to predict their locations using TargetP program (www.cbs.dtu.dk/services/TargetP). Of the identified proteins, 43 were predicted to be secreted with signal peptide sequences (Supplementary Table 2 and Supplementary Table 3). These proteins were also queried using SignalP 3.0 program, which is a web-based software containing two different algorithms (SignalP-NN and SignalP-HMM). We selected only the proteins harboring a signal peptide by both prediction algorithms^29^, which accounted for 32 proteins (Supplementary Table 2 and Supplementary Table 3). Proteins predicted to have signal peptide were cross-examined using ScanProsite, with PS00014 as a scan pattern to determine if the proteins were likely to be retained in the endoplasmic reticulum. Only five such proteins containing ER retention motif were excluded from the list of secreted proteins.

Glycosylphosphatidylinositol (GPI) linked proteins are secreted via the ER and Golgi apparatus to the extracellular space. In this study, no proteins were predicted to contain GPI anchors when examined with big-PI Plant Predictor program. There have been many reports of the existence of non-classical proteins in the extracellular space. Protein sequences were thus analyzed on the neural network server SecretomeP. The analysis predicted 63 LSPs (NN-scores >0.5; Supplementary Table 2 and Supplementary Table 3). Further, a critical review of literature, based on independent and orthogonal techniques, supports the presence of 81 of the identified proteins in the secretome. Taken together, 190 proteins that account for more than 90% were confirmed to be localized in the secretory fraction.

### Dynamic network of dehydration-responsive secreted proteins

It is increasingly clear that most environmental signals utilize the ECS as a conference point for their communication, albeit the role of secreted proteins in such cross-talk remains largely uninvestigated. To gain an insight into protein secretion during stress induced signal transduction, the identified proteins were grouped into 4 functional classes (Fig. 3C, Supplementary Table 2 and Supplementary Table 3). The criteria used to address the localization of the proteins, were based on their presence in other reported cell wall proteomes or secretomes in addition to the predicted biochemical and biological functions. Among the dehydration-responsive proteins in the secretome, the largest percentage was involved in ligands binding proteins (47%), followed by the proteins involved in metabolic process (20%), miscellaneous functions (20%) and catalytic activity (13%).

The communication between the cytoskeleton and the apoplast is one of the most characteristic features of cellular mechanism that allows cells to respond effectively to various extracellular signals, possibly through regulation of ROS^30^. Induced actin polymer formation is reported during disturbance of ROS homeostasis^31^. Protein kinase is a ubiquitous enzyme in eukaryotes and prokaryotes that catalyzes the transfer of the γ-phosphate from ATP to substrate auto-phosphorylation, thus contributing to downstream signaling by producing GTP for the activation of GTP-binding proteins. We observed dehydration induced regulation of protein kinase. Overexpression of protein kinase was previously reported to reduce the accumulation of ROS and provide tolerance against different abiotic stresses^32^. Probable serine/threonine-specific protein kinase, xylulose kinase-like, inactive purple acid phosphatase and protein phosphatase are likely candidates that might be involved in the signal transduction network that operates in the apoplast. Subtilisin-like protease, aspartic proteinase, cysteine proteinase and protease inhibitor were previously reported to be regulated differentially under stress conditions^33^. The enzymes related to secondary metabolism were also notable among the list of dehydration-responsive proteins like GMP synthase, an enzyme from the strictosidine biosynthesis pathway. The alkaloids have multiple functions, such as structural support, pigmentation, defense and signaling^34^. Most of the secondary metabolism related differentially expressed proteins, identified in this study, have been reported in the cell walls of different organisms. Cysteine synthase was earlier reported in cell wall of *Medicago*^35^. Spermidine synthase, serine hydroxymethyltransferase and aldolase were reported in the secondary cell wall of developing xylem tracheary elements^36^. Hexosaminidase, hydroquinone glucosyltransferase and dolichyl-diphosphooligosaccharide catalyzes an important trafficking step in cell wall modification. Tankyrase-2-like protein is required for synthesis of a variety of cellular constituents including cell wall polymers and glycoproteins. These results suggest that the differentially expressed secreted proteins may utilize the cell wall polysaccharides as a reservoir to produce sugar monomers, which maintain osmotic balance in plants under dehydration conditions.

During stress adaptation, protein degradation is necessary for the removal of abnormal or damaged proteins, and for altering the balance of proteins^34^. In this study, proteins involved in the degradation pathway were also identified. The 26S proteasome has been reported to be involved in ubiquitin-mediated turnover of misfolded proteins^37^. Since intact proteins are less sensitive to oxidation than misfolded proteins, protein chaperones are usually upregulated in response to various stresses. Different chaperones have been documented to play complementary and sometimes overlapping roles in protection of proteins. Many proteins from this class were also identified in the chickpea secretome *viz*., peptidyl-prolyl *cis-trans* isomerase, Hsp70 and 10 kDa chaperonin. Furthermore, nodal modulator, which is involved in nodal signaling and subsequent organization of axial structures, was also identified as differentially expressed protein.

The differential regulation of signaling molecules *viz*., GTP-binding protein and Ran GTPases, in the extracellular space suggests a complex signal transduction network. There are several reports that suggest extensive crosstalk among various environmental stress-responsive pathways^38^,^39^. The PR proteins form a novel class of proteins that play pleiotropic roles, both in abiotic and biotic stresses^40^. Induction of different isoforms of glucanases and thaumatin like-protein, among others, could be attributed to their multiple roles. In unstressed condition, the translation elongation factor (EF-Tu) catalyzes the GTP-dependent binding of the aminoacyl-tRNA to the ribosome during the elongation phase of protein synthesis. However, EF-Tu can act as a molecular chaperone during stress and might be involved in protein folding and protection^41^.

### Stress-induced non-classical secretion

The significance of non-classical secretion in plants, and the possible functions of LSPs are largely unknown. Most of the LSPs are related to stress response, and to explain their secretion, a number of alternate secretion mechanisms have been anticipated^42^,^43^. Leaderless secretion facilitates rapid release of stress-responsive proteins via Golgi/ER-independent pathway. It also allows a normally cytoplasmic protein to relocate to the ECS where it can perform alternate functions. One of the dehydration-responsive LSPs identified, in this study, is nucleolin, a highly conserved and ubiquitously expressed protein in eukaryotes^44^. It contains RGG repeats which participate in interactions with other proteins. Although it is highly abundant in the nucleus but often found in the plasma membrane and cytoplasm, where it is involved in numerous cellular processes. Lipid transfer protein is known to bind calcium intracellularly, but its role as an extracellular polypeptide signal has also been proposed^45^. It is likely that these proteins serve dual roles, both intracellular and extracellular, depending on environmental cues. A recent secretome study in mammalian cells revealed an intracellular cysteine protease as a regulator of non-classical protein secretion^46^. The quantitative study on *Arabidopsis* suspension culture confirmed that a relatively high number of LSPs are secreted into the ECS when treated with salicylic acid^47^. There are an increasing number of reports of individual proteins or enzyme activities that localize to the apoplast in response to stress despite the fact that such proteins lack signal peptide. We identified different isoforms of ubiquitin, proteasome and endopeptidases that lacked the signal peptide (Supplementary Table 3).

### Proteomic identification and cloning of CaRRP1, a non-classical secreted protein

**Figure 4:**
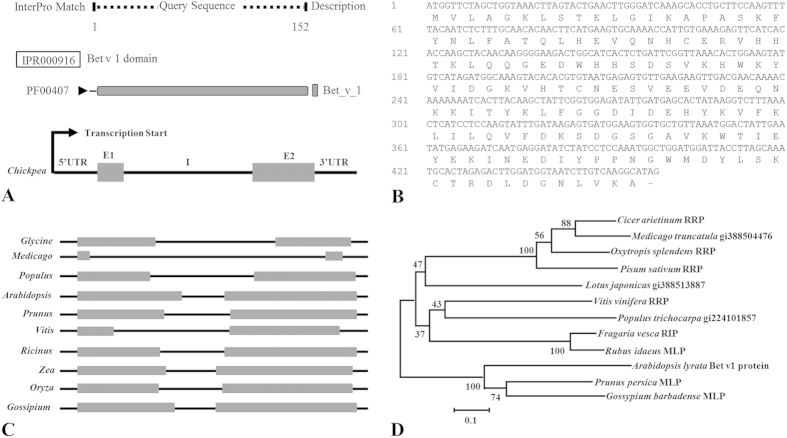
Structural and phylogenetic analysis of CaRRP1. (**A**) Domain analysis of CaRRP1 by InterPro Scan and schematic representation of its exon-intron organization. (**B**) Coding sequence of CaRRP1 along with deduced amino acids. (**C**) Genomic organization of CaRRP1 homologs in other plant species. (**D**) An unrooted phylogenetic tree showing evolutionary relationship of CaRRP1 with its orthologs. The phylogram was generated using the neighbor-joining algorithm of MEGA software, version 5.11. The numerical represents the bootstrap value. Scale bar indicates an evolutionary distance of 0.1 aa substitution per position in the sequence. E, exon; I, intron; and UTR, untranslated regions.

Screening of the secretome led to the identification of a non-classical secreted protein, henceforth designated CaRRP1 (ripening related protein). The domain analysis of CaRRP1 using InterProScan revealed the presence of Bet v 1 family domain (Fig. 4A). The Bet v 1 family proteins are proteins of unknown biological function that were first discovered in the plant latex and found to be upregulated during fruit ripening^48^. Most of these proteins were reported from dicotyledonous plants, and often referred to as cytokinin-specific binding proteins. The RRPs are of interest for several reasons: (i) change in their mRNA expression is accompanied in fruit ripening process; and (ii) the primary structure depicts significant homology to a yeast secretory protein implicated in signal transduction.

### Genomic organization of CaRRP1 and phylogenetic relationship

Genomic sequence comparison revealed the transcript size of *CaRRP1* to be 737 bp with coding region of 459 bp, and 43 bp 5′-UTR and 235 bp 3′-UTR. Further, the *CaRRP1* coding sequence is interrupted by a single intron (Fig. 4A). The *CaRRP1* encodes for a 152 amino acid protein with approximate molecular weight of 17.5 kDa and pI 5.9. The complete nucleotide sequence and deduced amino acid sequence are illustrated in Fig. 4B. The analysis of genomic organization in other taxa revealed that the RRP encoding genes include single intron with varied length ranging from the smallest in *Gossypium* to the longest in *Medicago* (Fig. 4C). To determine the evolutionary relationship, phylogenetic analysis was performed using representative RRPs from different taxa. The phylogram displayed two major Bet v 1 evolutionary groups (Fig. 4D). Members of *Arabidopsis* gene family were found to form distinct clades indicating an evolutionary divergence; however, CaRRP1 closely clustered with proteins from *Medicago*, possibly because both belong to family Fabaceae.

### Localization of leaderless CaRRP1

**Figure 5:**
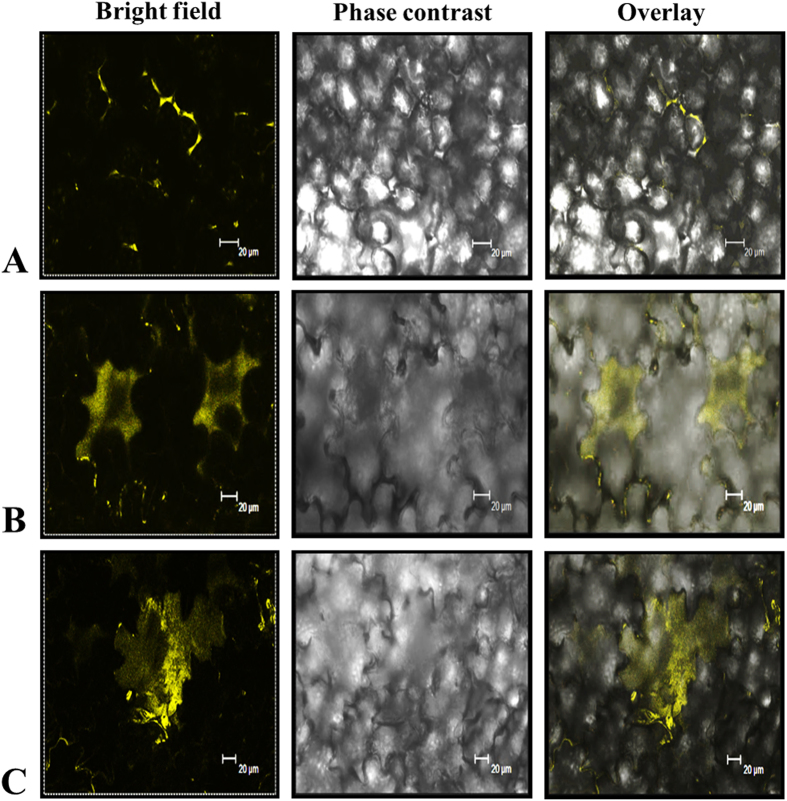
Subcellular localization and secretion analysis of leaderless protein CaRRP1. Localization was carried out in tobacco leaves using *Agrobacterium*-mediated transient expression without (**A,B**) and with (**C**) BFA (10 μg/mL), an inhibitor of secretion. Yellow fluorescence, visible light and merged images were taken from the epidermal cells, infiltrated with *Agrobacterium* suspension harboring the constructs encoding CaRRP1-YFP. Bars = 20 μm.

While *in silico* analysis using SignalP did not identify any clear targeting sequence, SecretomeP neural network programme could recognize the secretion of CaRRP1 via non-classical secretion. To validate the location, the coding region of *CaRRP1* was introduced into plant expression vector, harboring *YFP* reporter gene. The YFP fluorescence of the fusion protein in tobacco leaves was visualized following *Agrobacterium*-mediated transient expression. A time-dependent expression assay detected the YFP-CaRRP1 in the ECS (Fig. 5). The fluorescent signals were detected in the apoplast at 36 h of agro-infiltration (Fig. 5A), while there was an efflux of signals into the ECS after 48 h (Fig. 5B). To examine further, whether the recombinant protein translocates from cytoplasm to the extracellular space, the leaves were infiltrated with brefeldin A (BFA), an inhibitor of secretion^49^. The BFA treatment restricted the YFP fusion protein within the cells (Fig. 5C). These results and growing evidence for the unconventional secretion of proteins in non-plant systems support the possibility that alternative export pathways also exist in plants. The LSPs are known intracellular factors involved in various cellular activities in both plants^50^ and mammals^51^. Interestingly, once these proteins are secreted into the ECS as observed in mammals, they acquire new functions to behave as extracellular signaling molecules^42^. These findings strongly support the hypothesis that leaderless secretion might be ubiquitous in nature.

### Stress-induced expression of CaRRP1

**Figure 6:**
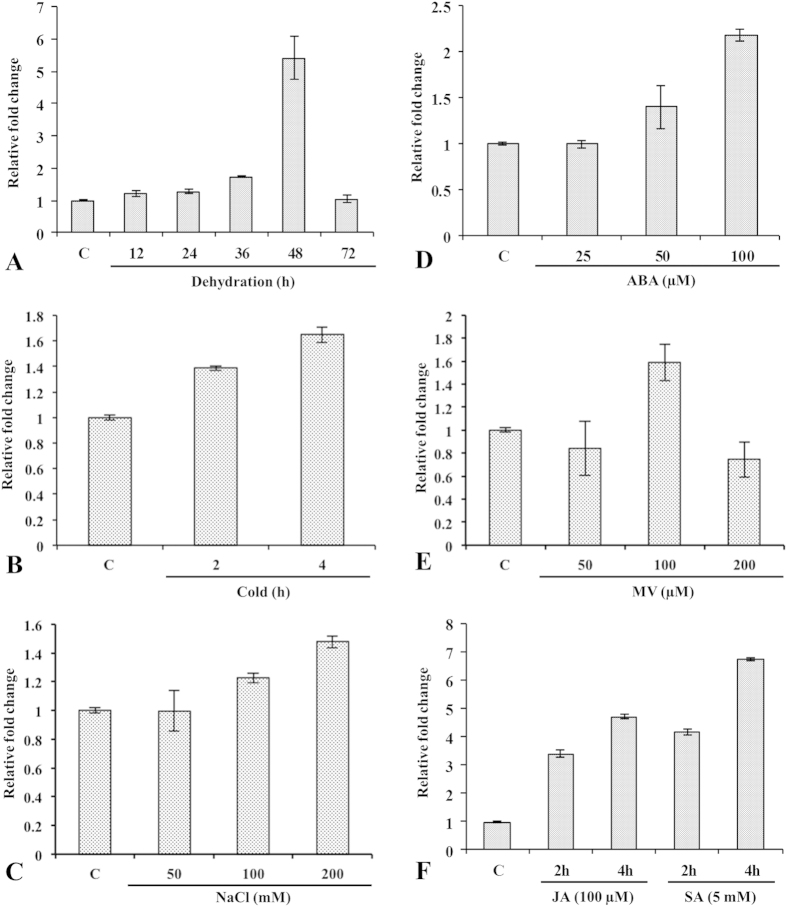
Determination of CaRRP1 transcript levels by qRT-PCR. Stress induced transcript accumulation was quantified in response to (**A**) dehydration, (**B**) cold, (**C**) NaCl, (**D**) ABA, (**E**) MV and (**F**) JA and SA. Relative fold change in mRNA level is shown on Y-axis. The internal standard EF1a was used for normalizing the qRT-PCR data. The experiments were accomplished in triplicates (n = 3). Vertical bars in the graphs indicate the S.E. C = control.

Regulation of gene expression at the transcriptional level plays a crucial role in the development and physiological status of plant. To investigate stress-responsive expression patterns of *CaRRP1* in chickpea, we carried out Northern blot analysis. The *CaRRP1* transcripts were highly upregulated under dehydration, displaying maximum accumulation at 48 h but dropped thereafter and reached the background level at 72 h (Supplementary Fig. 5). The quantitative accumulation of transcripts was further examined by qRT-PCR. The results showed that *CaRRP1* is responsive to multiple stresses such as dehydration, hypersalinity, cold, and treatment with methyl viologen (MV), jasmonic acid (JA) and salicylic acid (SA). The mRNA signals increased gradually from 12 to 48 h, but decreased at 72 h of dehydration (Fig. 6A). This expression pattern was similar as observed for their mRNA accumulation in response to other stresses such as cold (Fig. 6B), hypersalinity (Fig. 6C) and treatment with ABA (Fig. 6D) indicating that *CaRRP1* might participate in abiotic stress response possibly via ABA-dependent pathway. Interestingly, the expression of *CaRRP1* displayed increase of 2-fold in response to MV (Fig. 6E) while 4- to 6-fold upon JA and SA treatment (Fig. 6F) indicating its defense responses against diverse biotic stresses.

### Functional complementation analysis of CaRRP1 in yeast

**Figure 7:**
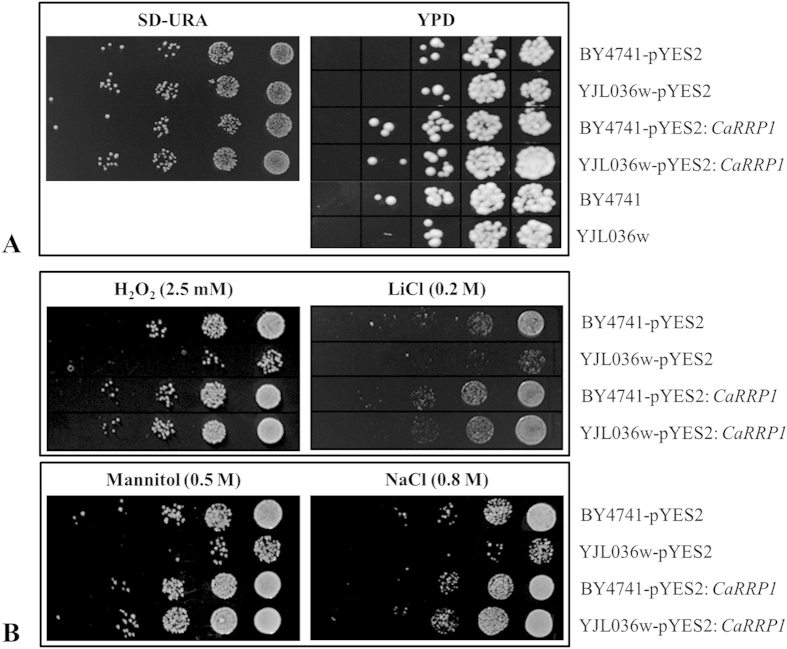
CaRRP1 complements the function of YJL036w in yeast. (**A**) Growth of wild-type (BY4741), BY4741:pYES2, YJL036w and complemented (YJL036w: CaRRP1) strains in unstressed condition, (**B**) in presence of 2.5 mM H_2_O_2_ and 0.2 M LiCl (upper panels), 0.5 M mannitol and 0.8 M NaCl (lower panels) in serial dilutions (10 to 10^−5^).

Over the years, complementation analysis in yeast led to the identification of more than 50 proteins involved in protein trafficking in the secretory pathway^52^. YJL036w is a sorting nexin protein involved in proteasome function^53^ and also functions in vesicular transport in yeast. YJL036w lacking yeast strains are defective in protein transport and exhibit aberrations in growth and appearance. To investigate the role of CaRRP1 in protein trafficking, we tested whether it could complement the *YJL036w* mutant. We used the YJL036w-deficient mutant for the complementation assay and monitored the growth of BY4741 (wild-type), *YJL036w* mutant and complemented (YJL036w:CaRRP1) strains in presence of various stressors that include 2.5 mM H_2_O_2_ for oxidative stress, 0.5 M mannitol for osmotic stress, 0.8 M NaCl and 0.2 M LiCl for hypersalinity. No significant difference in growth pattern of transformants, wild-type and mutants was observed either on SD-URA or YPD plates (Fig. 7A). However, the complemented strains grew rapidly and showed more tolerance to stress conditions when compared with the wild-type and mutant strains (Fig. 7B). These results suggest that CaRRP1 might participate in protein trafficking and restore normal growth in the mutant *YJL036w* indicating its putative stress-responsive role in secretory pathway.

## Conclusions

The extracellular fluid in higher plants plays critical roles in a multitude of biological and physiological processes such as signal perception, transduction, defense and stress-related responses^14^,^54^. Despite the biological importance, most research interest on plant responses to stress has been at the cellular level, while analysis of extracellular fluid remains secondary. To understand the underlying mechanism of stress tolerance, and to identify proteins for improving such important trait, dehydration-responsive secretome of chickpea was screened. This study represents the first effort to demarcate the molecular basis of the acquisition of dehydration tolerance in the suspension culture. The important considerations would be the method used to impose dehydration, the severity and duration of dehydration and how the observed responses fit into an overall strategy for better adaptation.

**Figure 8:**
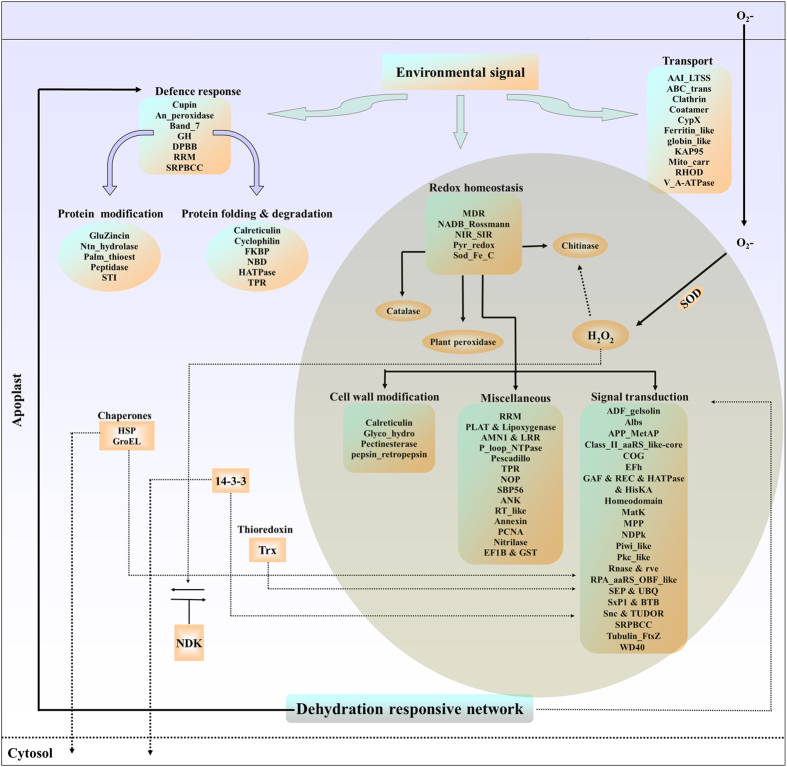
Prototype depicting diverse protein network in dehydration-responsive secretome of chickpea. Schematic diagram depicting involvement of dehydration-responsive proteins in different biological processes. Each box represents the list of secreted proteins categorized based on their participation in the corresponding metabolic activity.

The perception of dehydration condition is presumably regulated by cellular water status. The lipid peroxidation in terms of increased production of MDA after 48 h of dehydration seemingly resulted in induction of signaling proteins. Accumulation of compatible solutes is correlated with dehydration tolerance by protecting protein and membrane structure, regulating redox status, or acting as a scavenger of ROS^55^,^56^. Probable osmotic adjustment was observed as a result of proline accumulation at 72 h. The analysis of the secretome led to the identification of 215 differentially regulated proteins. Among the stress-responsive proteins, there were 18 unknown proteins that have not yet been characterized, and we could predict their association in stress response. Several dehydration-responsive proteins were identified that included functionally characterized proteins but previously not reported to be associated with stress. The results obtained have been illustrated as a representative prototype depicting diverse network putatively functional in the ECS (Fig. 8). Probable linkers between the cytoskeleton and ECS may relay biochemical/mechanical signals induced by dehydration stress to the cell interior. Protein modification pathway-related proteins were found to be induced that might aid the survival of plants under water-deficit condition. One of the LSPs, CaRRP1, a differentially regulated protein under dehydration was found to be involved in stress adaptation. These findings would bridge the gap between the events of ecophysiological experiments and comparative proteomics analysis in elucidating the molecular mechanisms by which plants sense and respond to such stress. The significance of the present study lies in the fact that abiotic stress responses are now being examined in the extracellular fluid, which remained largely uncharacterized. This catalogue of the differentially regulated proteins in the secretome would provide foundation for further functional studies in determining their precise biochemical roles in stress tolerance in plants.

## Methods

### Maintenance of SSCs and dehydration treatment

Seeds of chickpea (*Cicer arietinum* L. cv. JG-62) were surface-sterilized using 70% ethanol followed by 0.1% HgCl_2_ for 10 min and then rinsed 5–7 times with sterile distilled water. Surface-sterilized seeds were allowed to imbibe water for at least 16 h and kept aseptically in dark. The explants were prepared from root-shoot-cut embryo axes and inoculated on MS media (pH 5.8). The nascent calli were maintained at 25 ± 2 °C, under 16 h photoperiod (300 μmol m^−2^ s^−1^ light intensity). The friable embryogenic calli were used to initiate suspension cultures in liquid MS media as described earlier^20^. Dehydration stress was imposed after second sub-culturing by adding PEG 6000. The unstressed and the stressed cultures were maintained in parallel under same conditions. Secretory fractions of the SCCs, harvested at different time intervals, were used for downstream experiments.

### Determination of RWC and cell viability

The SCCs were weighed (fresh weight, FW) after filtration through pre-weighed 0.2 micron membrane. The cells were rehydrated until fully turgid, surface dried and weighed (turgid weight, TW), followed by oven drying at 80 °C for 48 h and reweighed (dry weight, DW). The RWC was calculated by the following formula^12^: RWC (%) = [(FW-DW/TW-FW) × 100]. Cell viability of suspension culture was determined and quantified as described earlier^20^ using Evans blue and FDA.

### Proline estimation, evaluation of electrolyte leakage and lipid peroxidation

Free proline content was measured as described earlier^12^. Proline content was determined as per the formula: proline (μg/g FW) = 36.6 × A_520_ × volume/2× FW. Electrolyte leakage was assayed by estimating the ions leaching from the SCCs. Aliquots of 20 ml SCCs in two sets were examined. The first set was kept at 25 ± 2 °C for 4 h and the conductivity (C1) was recorded. The second set was autoclaved followed by recording the conductivity (C2), and electrolyte leakage [1-(C1/C2) × 100] was calculated. In a separate experiment, lipid peroxidation was determined in terms of malondialdehyde (MDA) production^12^.

### Purification of secreted proteins from suspension culture

The suspension cultures were left untreated or treated with 10% PEG 6000 for 72 h and the secreted proteins were isolated^20^. In brief, the suspension culture was centrifuged at 3000 × g for 5 min followed by filtration using 0.45 μm Durapore membrane (Millipore). The resulting callus culture filtrate (CCF) was concentrated using Amicon Ultra-15 centrifugal filter unit (10 kDa cut-off each; Millipore) until the final protein concentration was >1 mg/ml. Further, the retentate was extracted as described earlier^8^. Also, proteins from calli were isolated^57^ with few modifications. Approximately, 300 mg calli were ground to powder in liquid nitrogen with 0.3% (w/w) polyvinylpolypyrollidone (PVPP) and powdered tissue was homogenized in homogenizing buffer [50 mM Tris-HCl (pH 8.2), 2 mM EDTA, 20% glycerol, 5 mM DTT and 2 mM PMSF]. The proteins were recovered as supernatant by centrifugation at 6000 × g for 10 min at 4 °C^58^. Protein samples were allowed to cool at 25 ± 2 °C, precipitated with 9 volumes of 100% chilled acetone overnight at −20 °C. The precipitates were recovered at 10,000 × g for 10 min at 4 °C. Protein pellets were washed twice with 80% acetone to remove excess SDS, air-dried and protein concentration was measured using the 2-D Quant Kit (GE Healthcare).

### Enzymatic assay of catalase

The catalase activity was determined calorimetrically as described earlier^58^. The reaction mixture was prepared by adding 10 μg protein, in 50 μl, to 940 μl of 70 mM potassium phosphate buffer (pH 7.5). Reaction was initiated by adding 10 μl of H_2_O_2_ (3% v/v), and decrease in absorbance at 240 nm was monitored for 5 min.

### Immunoblot screening

Immunoblotting was carried out by resolving secreted and calli proteins on 12.5% SDS-PAGE followed by electrotransfer onto nitrocellulose membrane (GE Healthcare). The blot was probed with anti-RbcL antibody (AS03037; Agrisera AB) at a dilution of 1:5000 in TBS. Immunoreactive protein was detected by incubation with alkaline phosphatase conjugated anti-rabbit IgG as secondary antibody (Sigma).

### Electrophoresis of secreted proteins

The proteins were resuspended in 2-D rehydration buffer [8 M urea, 2 M thiourea, 4% (w/v) CHAPS, 20 mM DTT, 0.5% (v/v) pharmalyte (pH 4–7) and 0.05% (w/v) bromophenol blue]. Isoelectric focusing was carried out with 150 μg protein in 250 μl buffer using 13 cm IPG strips (GE Healthcare) by in-gel rehydration method. Electrofocusing was performed using IPGphor system (GE Healthcare) at 20 °C for 30,000 Vh. In a separate experiment, the proteins were fractionated on 13 cm 1-DE. The electrophoresed proteins were visualized with MS-compatible silver staining (Bio-Rad Laboratories).

### Image acquisition and data analysis

Image acquisition was achieved by digitization of gel images with a Bio-Rad FluorS system. Quantitative and qualitative differences between the replicate 2-DE gels were analyzed using PDQuest version 7.2.0 (Bio-Rad Laboratories) followed by generation of reference image^12^. The replicate gels used for making the first level matchset had, at least, a correlation coefficient value of 0.8. In order to compare gels from individual time points, a second level matchset was created. A data matrix of high quality spots was constructed from unstressed and stressed samples for further analysis.

### In-gel digestion, mass spectrometry and bioinformatics analysis

The protein spots or lane (sliced into 29 gel pieces each of ~1.5 mm) were excised and subjected to trypsinolysis^58^. The peptides were analyzed using QSTAR Elite mass spectrophotometer (Applied Biosystem) coupled with an on-line Tempo nano-MDLC system. The acquired mass spectra were searched against the chickpea protein sequence available in chickpea genome annotation v.1.0^59^ (22893 sequences; 9330989 residues) using Mascot search engine (www.matrixscience.com). Proteins were assigned as identified if the MOWSE score was above the significance level. The function of proteins was assigned using protein function database Pfam (http://www.sanger.ac.uk/software/Pfam/) or Inter-Pro (http://www.ebi.ac.uk/interpro/). Further, the criteria used to assign the function of the proteins were based on other reports besides the predicted biochemical and biological functions by GO classification.

The presence and location of signal peptide cleavage sites were predicted by the SignalP 3.0 program (http://www.cbs.dtu.dk/services/SignalP). Proteins identified by SignalP were re-examined using ScanProsite (http://au.expasy.org/prosite/). To identify non-classical secreted proteins, proteins lacking *N*-terminal signal sequences were analyzed by SecretomeP (http://www.cbs.dtu.dk/services/SecretomeP/). The big-PI Plant Predictor program GPI (http://mendel.imp.ac.at/gpi/plants/gpi_plants.html) was used to identify potential GPI lipid anchors. The domain analysis was done by NCBI CDD (http://www.ncbi.nlm.nih.gov).

### Isolation of *CaRRP1*, sequence analyses and construction of phylogram

The cDNA fragment of *CaRRP1* was cloned into the pGEM-T vector (Promega) and the sequence identity was determined. The amino acid sequence, molecular weight and isoelectric points of CaRRP1 were obtained from ExPASy. Secondary structures, including the locations of the Bet v 1 domain were determined by InterProScan (http://www.ebi.ac.uk/interpro/). The SPIDEY (http://www.ncbi.nlm.nih.gov/spidey) program was used to determine the genomic organization of CaRRP1. The phylogram was constructed from amino acid alignment by neighbor-joining method (http://www.ebi.uk/Tool/clustalw/) using MEGA software version 5.11, with a bootstrap value of 100^60^.

### Quantitative real-time PCR

The chickpea seedlings were grown in green house, and maintained at 25 ± 2 °C and 50 ± 5% relative humidity under 16 h photoperiod (300 μM m^−2^ s^−1^ light intensity) as described previously^58^. Three-week-old seedlings were independently subjected to dehydration and hypersalinity treatment (50, 100 and 200 mM NaCl). The unstressed seedlings were also maintained in the same green house and tissues were collected every day during the course of the dehydration experiment, and finally pooled to normalize the growth and development effects, if any. ABA (25, 50 and 100 μM), MV (50, 100 and 200 μM), SA (5 mM) and JA (100 μM) treatment were accomplished by spraying solution on the leaflets. The low temperature treatment was given by keeping the seedlings at 4 °C. The harvested tissues were instantly frozen in liquid nitrogen and stored at −80 °C. Total RNA was isolated from 3-week-old seedlings using the TriPure reagent (Invitrogen). cDNA was prepared using SuperScript^®^ VILO^TM^ cDNA Synthesis Kit (Invitrogen). The qRT-PCR assays were performed with the ABI PRISM 7700 sequence detection system (Applied Biosystems) using SYBR Green PCR Master mix in a final volume of 20 μl including cDNA template and appropriate primers (Supplementary Table 4).

### Localization of CaRRP1

The coding region of *CaRRP1* was amplified by PCR using gene-specific primers (Supplementary Table 4) and cloned into pENTR-D/TOPO followed by recombination into pGWB441, and introduced into *Agrobacterium* strain GV3101. The cells were infiltrated into tobacco leaves for the transient expression of CaRRP1-YFP fusion protein. The YFP fluorescence was detected with TCS SP2 confocal system (Leica, Germany) and the images were captured 2–3 days after infiltration.

### Functional complementation in yeast

The yeast mutant for vesicular transport *YJL036w* [BY4741; MATa; his3Δ1; leu2Δ0; met15Δ0; ura3Δ0; YJL036w:: kanMX4] and the background strain BY4741 [MATa; his3Δ 1; leu2Δ0; met15Δ0; ura3Δ0] were transformed with pYES2 and the recombinant *pYES2:CaRRP1* construct independently. Overnight cultures were grown in SD-URA and serial dilutions [OD_600_ ~ 0.1] were plated. The plates were incubated at 30 °C for 2 days. Growth assays were performed by using serial dilution of cultures in the respective media containing various stress-inducing chemicals (2.5 mM H_2_O_2_, 0.5 M mannitol, 0.8 M NaCl and 0.2 M LiCl).

## Additional Information

**How to cite this article**: Gupta, S. *et al.* Secretome analysis of chickpea reveals dynamic extracellular remodeling and identifies a Bet v1-like protein, CaRRP1 that participates in stress response. *Sci. Rep.* **5**, 18427; doi: 10.1038/srep18427 (2015).

## Supplementary Material

Supplementary Information

## References

[b1] Oparka K. J. Getting the message across: how do plant cells exchange macromolecular complexes? Trends Plant Sci. 9, 33–40 (2004). 14729217 10.1016/j.tplants.2003.11.001

[b2] Bortoluzzi S., Scannapieco P., Cestaro A., Danieli G. A. & Schiaffino S. Computational reconstruction of the human skeletal muscle secretome. Proteins 62, 776–792 (2006). 16342272 10.1002/prot.20803

[b3] Agrawal G. K., Jwa N. S., Lebrun M. H., Job D. & Rakwal R. Plant secretome: unlocking secrets of the secreted proteins. Proteomics 10, 799–827 (2010). 19953550 10.1002/pmic.200900514

[b4] Alexandersson E., Ali A., Resjo S. & Andreasson E. Plant secretome proteomics. Front. Plant Sci. 4, 9 (2013). 23378846 10.3389/fpls.2013.00009 PMC3561728

[b5] Shibaoka H. J. The use of tobacco BY-2 cells for the studies of the plant cytoskeleton. Plant Res. 3, 3–15 (1993).

[b6] Anderson N. L. & Anderson N. G. The human plasma proteome: history, character, and diagnostic prospects. Mol. Cell. Proteomics 1, 845–867 (2002). 12488461 10.1074/mcp.r200007-mcp200

[b7] Lewis J. A., Dennis W. E., Hadix J. & Jackson D. A. Analysis of secreted proteins as an *in vitro* model for discovery of liver toxicity markers. J. Proteome Res. 9, 5794–5802 (2010). 20822094 10.1021/pr1005668

[b8] Kaffarnik F. A., Jones A. M., Rathjen J. P. & Peck S. C. Effector proteins of the bacterial pathogen *Pseudomonas syringae* alter the extracellular proteome of the host plant, *Arabidopsis thaliana*. Mol. Cell. Proteomics 8, 145–156 (2009). 18716313 10.1074/mcp.M800043-MCP200

[b9] Wang W., Vinocur B. & Altman A. Plant responses to drought, salinity and extreme temperatures: towards genetic engineering for stress tolerance. Planta 218, 1–14 (2003). 14513379 10.1007/s00425-003-1105-5

[b10] Bray E. A. Genes commonly regulated by water-deficit stress in *Arabidopsis thaliana*. J. Exp. Bot. 55, 2331–2341 (2004). 15448178 10.1093/jxb/erh270

[b11] Chaves M. M. & Oliveira M. M. Mechanisms underlying plant resilience to water deficits: prospects for water-saving agriculture. J. Exp. Bot. 55, 2365–2384 (2004). 15475377 10.1093/jxb/erh269

[b12] Bhushan D. *et al.* Comparative proteomics analysis of differentially expressed proteins in chickpea extracellular matrix during dehydration stress. Mol. Cell. Proteomics 6, 1868–1884 (2007). 17686759 10.1074/mcp.M700015-MCP200

[b13] Xiong L. M., Schumaker K. S. & Zhu J. K. Cell signaling during cold, drought, and salt stress. Plant Cell 14, S165–S183 (2002). 12045276 10.1105/tpc.000596 PMC151254

[b14] Shinozaki K. & Yamaguchi-Shinozaki K. Gene networks involved in drought stress response and tolerance. J. Exp. Bot. 58, 221–227 (2007). 17075077 10.1093/jxb/erl164

[b15] Riccardi F., Gazeau P., de Vienne D. & Zivy M. Protein changes in response to progressive water deficit in maize: Quantitative variation and polypeptide identification. Plant Physiol. 117, 1253–1263 (1998). 9701581 10.1104/pp.117.4.1253 PMC34889

[b16] Kusumawati L., Imin N. & Djordjevic M. A. Characterization of the secretome of suspension cultures of *Medicago* species reveals proteins important for defense and development. J. Proteome Res. 7, 4508–4520 (2008). 18781796 10.1021/pr800291z

[b17] Brunner Y., Schvartz D., Coute Y. & Sanchez J. C. Proteomics of regulated secretory organelles. Mass Spectrom. Rev. 28, 844–867 (2009). 19301366 10.1002/mas.20211

[b18] Krause C., Richter S., Knoll C. & Jurgens G. Plant secretome - From cellular process to biological activity. Biochim. Biophys. Acta 1834, 2429–2441 (2013). 23557863 10.1016/j.bbapap.2013.03.024

[b19] Blum A. Drought resistance, water-use efficiency, and yield potential-are they compatible, dissonant, or mutually exclusive? Austral. J. Agr. Res. 56, 1159–1168 (2005).

[b20] Gupta S. *et al.* Characterization of the secretome of chickpea suspension culture reveals pathway abundance and the expected and unexpected secreted proteins. J. Proteome Res. 10, 5006–5015 (2011). 21923182 10.1021/pr200493d

[b21] Epstein E. *et al.* Saline culture of crops: A genetic approach. Science 210, 399–404 (1980). 17837407 10.1126/science.210.4468.399

[b22] Macrobbie E. A. C. Ion uptake. University of California Press, Berkeley 676–713 (1974).

[b23] Huang W. L. & Liu F. Carbohydrate metabolism in rice during callus induction and shoot regeneration induced by osmotic stress. Bot. Bull. Acad. Sci. 43, 107–113 (2002).

[b24] Rains D. W. Plant tissue and protoplast culture: applications to stress physiology and biochemistry. Cambridge University Press, Cambridge, UK 181–196 (1989).

[b25] Singh T. N., Paley L. G. & Aspinall D. Stress metabolism in nitrogen metabolism and growth in the barley plant during water stress. Aust. J. Biol. Sci. 26, 45–56 (1973).

[b26] Handa S., Handa A. K., Hasegawa P. M. & Bressan R. A. Proline accumulation and the adaptation of cultured plant cells to water stress. Plant Physiol. 80, 938–945 (1986). 16664745 10.1104/pp.80.4.938 PMC1075233

[b27] Bartholomew D. M., Bartley G. E. & Scolnik P. A. Abscisic acid, control of *rbcS* and *cub* transcription in tomato leaves. Plant Physiol. 96, 291–296 (1991). 16668167 10.1104/pp.96.1.291 PMC1080748

[b28] Bayer E. M. *et al.* *Arabidopsis* cell wall proteome defined using multidimensional protein identification technology. Proteomics 6, 301–311 (2006). 16287169 10.1002/pmic.200500046

[b29] Bendtsen J. D., Jensen L. J., Blom N., von Heijne G. & Brunak S. Feature-based prediction of non-classical and leaderless protein secretion. Protein Eng. Des. Sel. 17, 349–356 (2004). 15115854 10.1093/protein/gzh037

[b30] Baluska F., Samaj J., Wojtaszek P., Volkmann D. & Menzel D. Cytoskeleton-plasma membrane-cell wall continuum in plants. Emerging links revisited. Plant Physiol. 133, 482–491 (2003). 14555777 10.1104/pp.103.027250 PMC523875

[b31] Livanos P., Galatis B., Quader H. & Apostolakos P. Disturbance of reactive oxygen species homeostasis induces atypical tubulin polymer formation and affects mitosis in root-tip cells of *Triticum turgidum* and *Arabidopsis thaliana*. Cytoskeleton 69, 1–21 (2012). 21976360 10.1002/cm.20538

[b32] Moon H. *et al.* NDP kinase 2 interacts with two oxidative stress-activated MAPKs to regulate cellular redox state and enhances multiple stress tolerance in transgenic plants. Proc. Natl. Acad. Sci. USA 100, 358–363 (2003). 12506203 10.1073/pnas.252641899 PMC140977

[b33] Fu Y., Zhao W. & Peng Y. Induced expression of oryzain alpha gene encoding a cysteine proteinase under stress conditions. J. Plant Res. 120, 465–469 (2007). 17404686 10.1007/s10265-007-0080-5

[b34] Coux O., Tanaka K. & Goldberg A. L. Structure and functions of the 20S and 26S proteasomes. Annu. Rev. Biochem. 65, 801–847 (1996). 8811196 10.1146/annurev.bi.65.070196.004101

[b35] Watson B. S., Lei Z., Dixon R. A. & Sumner L. W. Proteomics of *Medicago sativa* cell walls. Phytochemistry 65, 1709–1720 (2004). 15276432 10.1016/j.phytochem.2004.04.026

[b36] Anderson L. E. & Carol A. A. Seven enzymes of carbon metabolism, including three Calvin cycle isozymes, are present in the secondary cell wall thickenings of the developing xylem tracheary elements in pea leaves. Int. J. Plant Sci. 165, 243–256 (2004).

[b37] DiDonato J. *et al.* Mapping of the inducible IkappaB phosphorylation sites that signal its ubiquitination and degradation. Mol. Cell. Biol. 16, 1295–1304 (1996). 8657102 10.1128/mcb.16.4.1295 PMC231113

[b38] Yamaguchi-Shinozaki K. & Shinozaki K. A novel cis-acting element in an *Arabidopsis* gene is involved in responsiveness to drought, low-temperature, or high-salt stress. Plant Cell 6, 251–264 (1994). 8148648 10.1105/tpc.6.2.251 PMC160431

[b39] Cheong Y. H. *et al.* Transcriptional profiling reveals novel interactions between wounding, pathogen, abiotic stress and hormonal responses in *Arabidopsis*. Plant Physiol. 129, 661–677 (2002). 12068110 10.1104/pp.002857 PMC161692

[b40] Mousavi A. & Hotta Y. Glycine-rich proteins: a class of novel proteins. Appl. Biochem. Biotechnol. 120, 169–174 (2005). 15767691 10.1385/abab:120:3:169

[b41] Caldas T. D., Yaagoubi A. E. & Richarme G. Chaperone properties of bacterial elongation factor EF-Tu. J. Biol. Chem. 273, 11478–11482 (1998). 9565560 10.1074/jbc.273.19.11478

[b42] Nickel W. & Seedorf M. Unconventional mechanisms of protein transport to the cell surface of eukaryotic cells. Annu. Rev. Cell Dev. Biol. 24, 287–308 (2008). 18590485 10.1146/annurev.cellbio.24.110707.175320

[b43] Radisky D. C., Stallings-Mann M., Hirai Y. & Bissell M. J. Single proteins might have dual but related functions in intracellular and extracellular microenvironments. Nat. Rev. Mol. Cell Biol. 10, 228–234 (2009). 19190671 10.1038/nrm2633 PMC2746016

[b44] Mongelard F. & Bouvet P. Nucleolin: a multiFACeTed protein. Trends Cell Biol. 17, 80–86 (2007). 17157503 10.1016/j.tcb.2006.11.010

[b45] Neumann S., Ziv E., Lantner F. & Schechter I. Regulation of HSP70 gene expression during the life cycle of parasitic helminth *Schistosoma mansoni*. Eur. J. Biochem. 212, 589–596 (1993). 8444195 10.1111/j.1432-1033.1993.tb17697.x

[b46] Keller M., Rüegg A., Werner S. & Beer H.-D. Active caspase-1 is a regulator of unconventional protein secretion. Cell 132, 818–831 (2008). 18329368 10.1016/j.cell.2007.12.040

[b47] Cheng F. Y., Blackburn K., Lin Y. M., Goshe M. B. & Williamson J. D. Absolute protein quantification by LC/MS^E^ for global analysis of salicylic acid-induced plant protein secretion responses. J. Proteome Res. 8, 82–93 (2009). 18998720 10.1021/pr800649s

[b48] Fujimoto Y. *et al.* Purification and cDNA cloning of cytokinin-specific binding protein from mung bean (*Vigna radiata*). Eur. J. Biochem. 258, 794–802 (1998). 9874249 10.1046/j.1432-1327.1998.2580794.x

[b49] Staehelin L. A. & Driouich A. Brefeldin A effects in plants (are different Golgi responses caused by different sites of action?). Plant Physiol. 114, 401–403 (1997). 12223714 10.1104/pp.114.2.401 PMC158318

[b50] Montrichard F., Alkhalfioui F., Yano H. & Vensel W. H. Thioredoxin targets in plants: the first 30 years. J. Proteomics 72, 452–474 (2009). 19135183 10.1016/j.jprot.2008.12.002

[b51] Arner E. S. & Holmgren A. Physiological functions of thioredoxin and thioredoxin reductase. Eur. J. Biochem. 267, 6102–6109 (2000). 11012661 10.1046/j.1432-1327.2000.01701.x

[b52] Bonifacino J. S. Vesicular transport earns a Nobel. Trends Cell Biol. 24, 3–5 (2014). 24373306 10.1016/j.tcb.2013.11.001 PMC4788104

[b53] Worby C. A. & Dixon J. E. Sorting out the cellular functions of sorting nexins. Nat. Rev. Mol. Cell Biol. 3, 919–931 (2002). 12461558 10.1038/nrm974

[b54] Wardhan V. *et al.* Overexpression of CaTLP1, a putative transcription factor in chickpea (*Cicer arietinum* L.), promotes stress tolerance. Plant Mol. Biol. 79, 479–93 (2012). 22644439 10.1007/s11103-012-9925-y

[b55] Hare P. D., Cress W. A. & van Staden J. Dissecting the roles of osmolyte accumulation during stress. Plant Cell Environ. 21, 535–553 (1998).

[b56] Hincha D. K. & Hagemann M. Stabilization of model membranes during drying by compatible solutes involved in the stress tolerance of plants and microorganisms. Biochem. J. 383, 277–283 (2004). 15225123 10.1042/BJ20040746 PMC1134068

[b57] Schiltz S. *et al.* Maps of vegetative tissues in pea. An investigation of nitrogen mobilization from leaves during seed filling proteome reference. Plant Physiol. 135, 2241- 2260 (2004). 15299134 10.1104/pp.104.041947 PMC520794

[b58] Bhushan D. *et al.* Extracellular matrix proteome of chickpea (*Cicer arietinum* L.) illustrates pathway abundance, novel protein functions and evolutionary perspect. J. Proteome Res. 5, 1711–1720 (2006). 16823979 10.1021/pr060116f

[b59] Varshney R. K. *et al.* Draft genome sequence of chickpea (*Cicer arietinum*) provides a resource for trait improvement. Nat. Biotechnol. 31, 240–246 (2013). 23354103 10.1038/nbt.2491

[b60] Yang Z. *et al.* Genomewide comparative phylogenetic and molecular evolutionary analysis of tubby-like protein family in *Arabidopsis*, rice, and poplar. Genomics 92, 246–253 (2008). 18620041 10.1016/j.ygeno.2008.06.001

