# Supplementary material for: Secretome analysis of chickpea reveals dynamic extracellular remodeling and identifies a Bet v1-like protein, CaRRP1 that participates in stress response
Source: Sci Rep. 2015 Dec 18;5:18427. doi: 10.1038/srep18427 (PMC4683448; doi:10.1038/srep18427)
Supplement: Supplementary Information [file srep18427-s1.pdf]

Secretome analysis of chickpea reveals dynamic extracellular remodeling and identifies a Bet v1-like protein, CaRRP1 that participates in stress response

Sonika Gupta[§], Vijay Wardhan[§], Amit Kumar, Divya Rathi, Aarti Pandey, Subhra Chakraborty* and Niranjan Chakraborty*

National Institute of Plant Genome Research, Aruna Asaf Ali Marg, New Delhi-110067, India.

Running title: Role of secreted proteins in extracellular remodeling and stress response

[§]These authors contributed equally.

Corresponding author

*****Dr. Niranjan Chakraborty
National Institute for Plant Genome Research
Aruna Asaf Ali Marg, New Delhi-110067, India.
E-mail: nchakraborty@nipgr.res.in

Tel: 00-91-11-26735178
Fax: 00-91-11-26741658

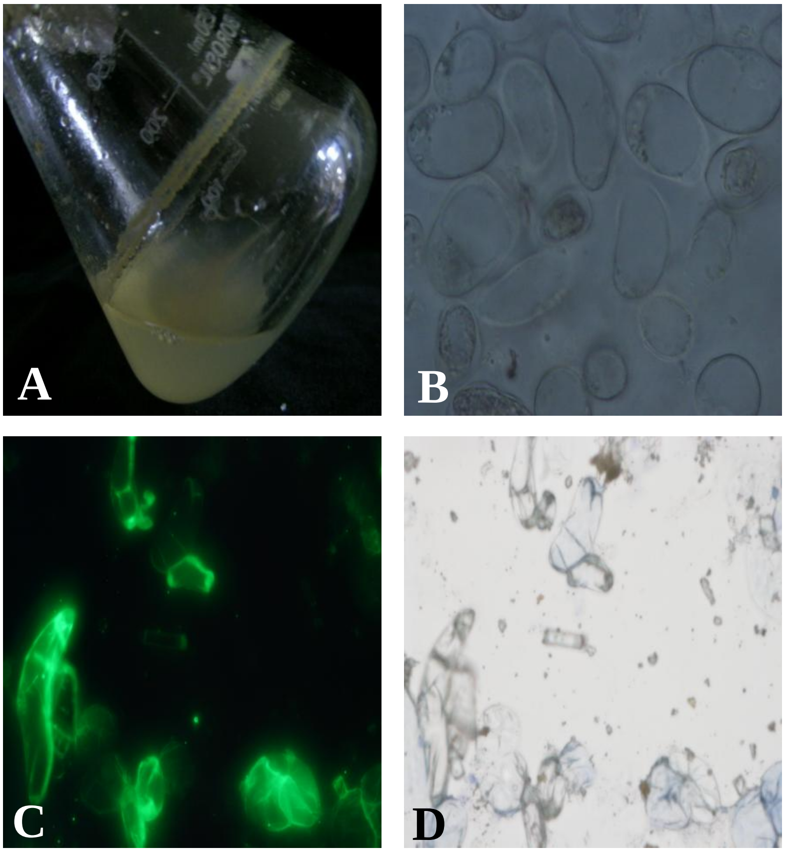

Supplementary Figure 1. Dehydration-induced morphological and ultrastructural changes in suspension culture. (A) Morphological analysis of chickpea suspension culture. (B) Suspension-cultured cells were scored under bright field illumination. (C) Fluorescence image of the cells stained with FDA to monitor cell viability and emitting green fluorescence. (D) Determination of necrosis in the culture by counter staining with Evan’s blue dye.

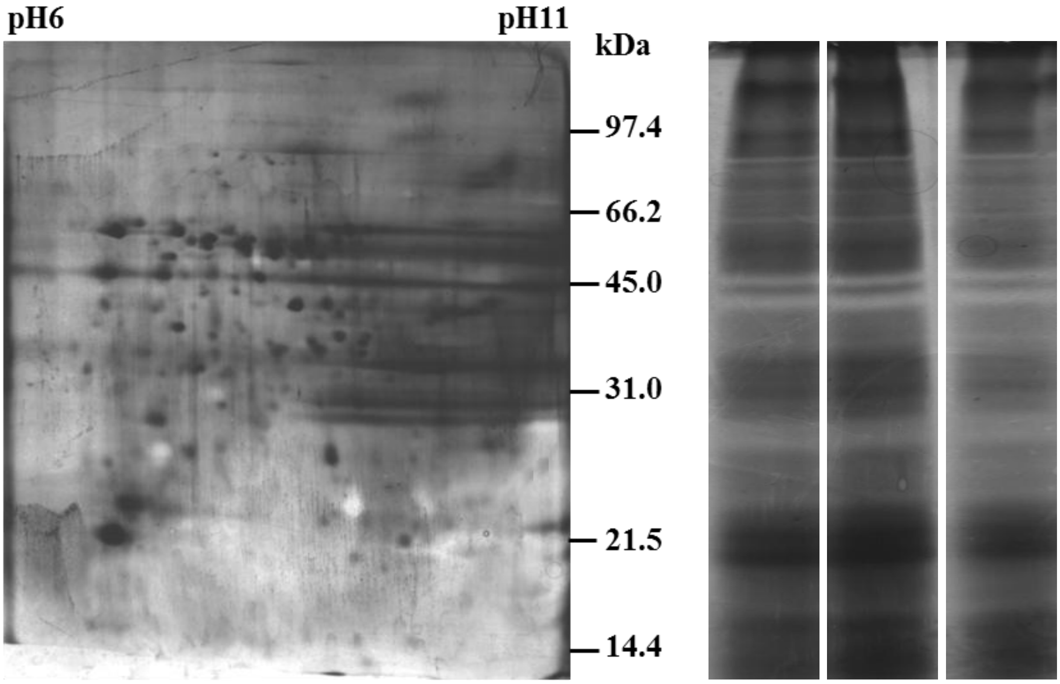

Supplementary Figure 2. Resolution of secreted proteins on 1- and 2-DE. (A) Secreted proteins were electrofocused on 13 cm IPG strip (pH 4-7), and separated onto 12.5% SDS-PAGE. (B) Alternatively, the proteins were resolved onto 13 cm 1-DE. The gels were stained by Silver Stain Plus® kit and the spots were visualized as described in Methods.

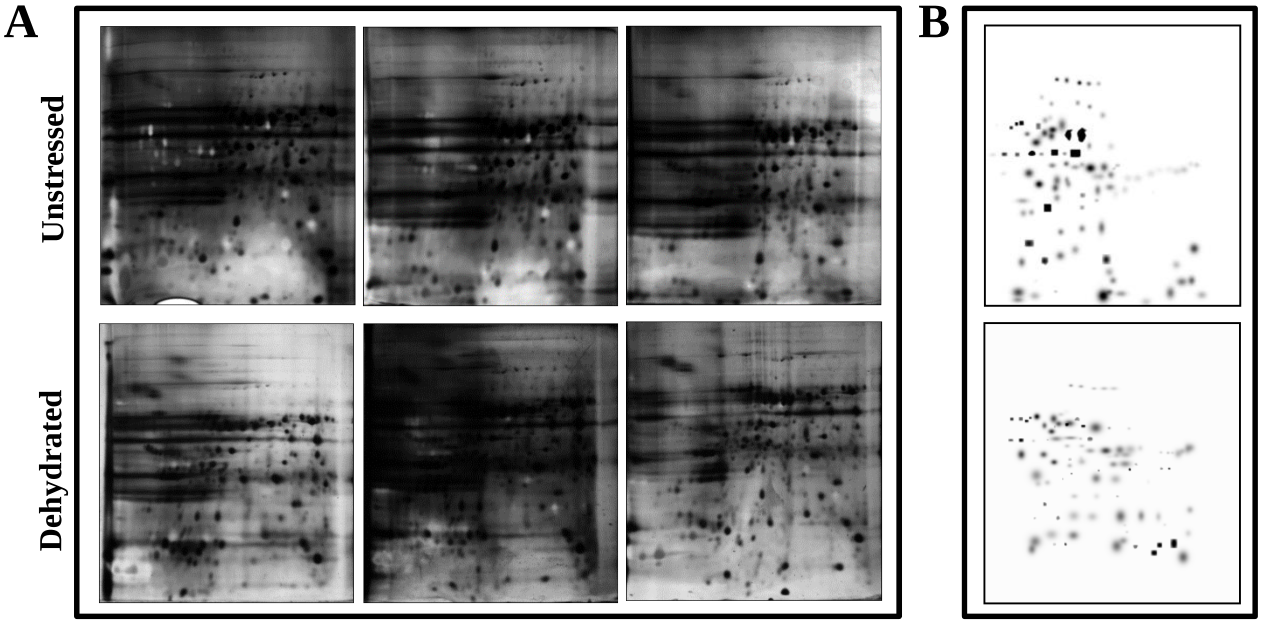

Supplementary Figure 3. Dehydration-responsive comparative secretome and the representative 2-DE gels. (A) Equal amounts (150 µg) of protein from unstressed and treated callus culture were resolved by 2-DE. The experiment was carried out in 3 replicate gels. (B) The replicate gels were combined computationally using PDQuest software (version 7.2.0) to generate the reference gel.

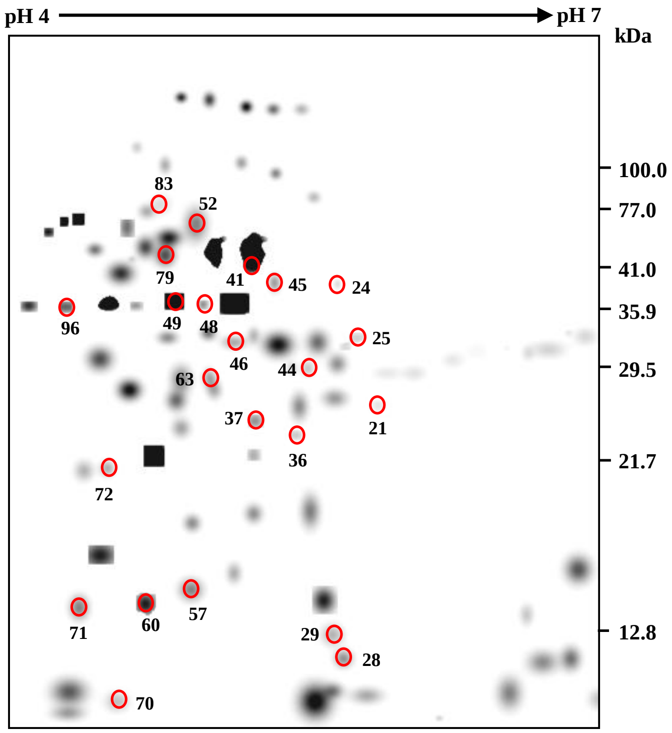

Supplementary Figure 4. Quantitative analysis of changes in protein expression. PDQuest software version 7.2.0 was used to assemble the match sets where replicate gels were compared. The higher level match set of the protein spots, detected by 2-DE, was created *in silico* by combining data from unstressed and dehydrated secretome. The identified secreted proteins are encircled.

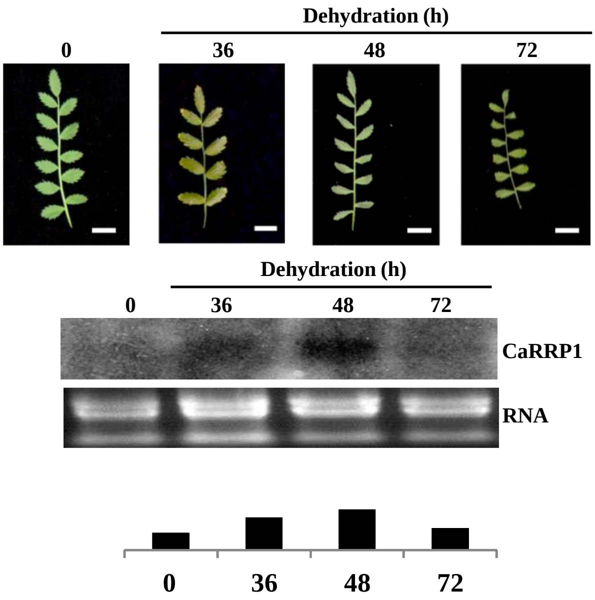

Supplementary Figure 5. Transcript analyses of *CaRRP1* in response to dehydration stress. The total RNA from different tissues, as shown in the upper panel, was fractionated on 1% (w/v) agarose gel, blotted onto nylon membrane, and probed with ^{32}P-labeled 0.459 kb *CaRRP1* cDNA. Ethidium bromide-stained rRNAs (bottom section) shows uniform loading and RNA quality. The graphs are indicative of the extent of expression in terms of band density of *CaRRP1*.

Supplementary Table 1. Reproducibility of 2-DE gels.

Time point	Total no. of spots[a]		High quality spots[b]		Reproducibility (%)
	Average	SE[c]	Average	SE[c]	
Control	110	1.54	104	2.02	94.54
72 h	105	2.67	100	3.24	95.23
Total	215	-	204	-	94.88

a) Average number of spots present in three replicate gels of each time point.
b) Spots having quality score more than 30 assigned by PDQuest (Ver.7.2.0).
c) SE represents standard error for the replicate gels.

Supplementary Table 2. List of identified secreted proteins in chickpea suspension culture by 2DE.

Accession No.[a]	GI	Protein identification	Mass	PI	Score	Queries matched	Length	SignalP (Cleavage site)	TargetP[b]	SecretomeP	GO[c]
Ca_21532	502089588	PREDICTED: uncharacterized protein	37983	7.22	34	1	339	_	others	Yes	_
Ca_21861	502086996	PREDICTED: uncharacterized protein LOC101495708	24446	6.98	34	1	215	_	others	No	_
Ca_20848	828333798	PREDICTED: uncharacterized protein LOC101514110	61347	9.11	37	1	540	_	others	Yes	Transcriptional regulation
Ca_24653	828333798	PREDICTED: uncharacterized protein LOC101514110	60996	9.18	37	1	535	_	others	Yes	Transcriptional regulation
Ca_01964	502161374	PREDICTED: ferredoxin--NADP reductase, leaf isozyme, chloroplastic	41056	8.37	69	2	364	YES(1-16)	others	No	electron transport; signalling; defence response; oxidoreductase;
Ca_11140	525313464	glucan endo-1,3-beta-glucosidase-like precursor	35630	5.5	34	1	331	YES(1-22)	S	Yes	metabolic process; defence response
Ca_00767	828301493	PREDICTED: LOW QUALITY PROTEIN: fructose-bisphosphate aldolase, cytoplasmic isozyme-like	37151	5.81	55	1	343	_	others	No	glycolytic processes; transcriptional regulation; cell differentiation
Ca_09753	502099695	PREDICTED: fructose-bisphosphate aldolase, cytoplasmic isozyme 1	41328	5.93	55	1	381	YES(1-28)	S	No	metabolic process; transcriptional regulation; cell differentiation
Ca_20130	502122153	PREDICTED: fructose-bisphosphate aldolase, cytoplasmic isozyme-like	38726	5.96	55	1	358	_	others	No	metabolic process; transcriptional regulation; cell differentiation
Ca_18250	828333798	PREDICTED: uncharacterized protein	52880	9.23	33	1	465	_	others	Yes	transcriptional regulation
Ca_24380	502089588	PREDICTED: uncharacterized protein	58063	8.58	34	1	526	_	others	Yes	transcriptional regulation
Ca_18117	828293899	PREDICTED: uncharacterized protein	19226	9.63	32	1	163	_	others	No	_

[a] Accession no. as in MSDB database. [b] S in the column indicates the proteins predicted in secretory fraction. [c] GO program used for the prediction of function of protein ontologically.

Supplementary Table 3. List of identified secreted proteins in chickpea suspension culture by 1DE.

Accession No.[a]	GI	Protein identification	Mass	PI	Score	Queries matched	Length	SignalP (Cleavage site)	TargetP[b]	SecretomeP	GO[c]	Reported in Literature[d]
Ca_23764	148612111	14-3-3-like protein [Cicer arietinum]	29347	4.71	953	24	260	_	others	No	protein binding	+
Ca_15135	358347278	Protein tolB [Medicago truncatula]	67711	5.93	153	3	599	_	others	Yes	biological_process	
Ca_19358	502102461	beta-hexosaminidase 1-like [Cicer arietinum]	20315	8.99	105	1	182	_	S	Yes		
Ca_19340	502104077	high mobility group B protein 1-like isoform X1 [Cicer arietinum]	17082	9.7	49	2	154	_	others	Yes		
Ca_01465	502133153	uncharacterized protein LOC101490938 [Cicer arietinum]	295937	8.84	35	6	2630	_	S	Yes		
Ca_12687	502135738	uncharacterized protein LOC101497537 [Cicer arietinum]	20748	4.78	100	3	187	YES(1-24)	S	Yes	protein binding	
Ca_05047	502141618	uncharacterized protein LOC101508154 [Cicer arietinum]	25945	7.01	94	4	232	YES(1-24)	S	Yes		
Ca_05262	502142141	uncharacterized protein LOC101501714 [Cicer arietinum]	32280	9.08	264	7	302	YES(1-25)	S	Yes		
Ca_13439	502147774	uncharacterized protein At2g34160-like [Cicer arietinum]	14450	5.42	159	4	133	_	others	No	nucleic acid binding	
Ca_03228	502150299	uncharacterized protein LOC101505055 [Cicer arietinum]	85459	4.96	33	2	760	_	others	No		
Ca_12374	502154568	uncharacterized protein LOC101513427 [Cicer arietinum]	29634	6.16	45	2	279	_	others	Yes	nucleic acid binding	
Ca_21370	502158394	uncharacterized protein LOC101504873 [Cicer arietinum]	15390	4.32	106	3	140	_	others	No	binding	
Ca_09210	568215132	uncharacterized protein LOC102577793 [Solanum tuberosum]	14838	9.89	65	4	130	_	others	No	structural molecule activity	
Ca_22494	502103080	hydroquinone glucosyltransferase-like [Cicer arietinum]	52835	5.94	142	3	475	_	others	No	metabolic process	
Ca_06137	502106726	vicilin-like [Cicer arietinum]	51865	5.73	340	8	455	YES(1-23)	S	Yes	molecular_function	
Ca_06122	502105576	regulator of ribonuclease-like protein 2-like [Cicer arietinum]	18062	5.39	216	6	166	_	others	No	enzyme regulator activity	
Ca_00356	502078151	ras-related protein RABA1f-like [Cicer arietinum]	24223	5.34	199	6	217	_	others	No	nucleotide binding	+
Ca_09533	502104677	bifunctional dihydroflavonol 4-reductase/flavanone 4-reductase-like isoform X2 [Cicer arietinum]	36544	5.73	73	2	326	_	others	No	catalytic activity	+
Ca_04063	502136652	nascent polypeptide-associated complex subunit alpha-like protein 2-like [Cicer arietinum]	24892	4.3	118	2	228	_	others	Yes	protein binding	+
Ca_08225	502107167	spermidine hydroxycinnamoyl transferase-like [Cicer arietinum]	53810	8.29	76	4	472	_	_	Yes	transferase activity	
Ca_08156	502107484	COP9 signalosome complex subunit 2-like [Cicer arietinum]	51719	6.11	60	2	439	_	others	No	protein binding	
Ca_07193	502108251	strictosidine synthase 1-like [Cicer arietinum]	40532	5.95	66	2	369	_	S	No	catalytic activity	
Ca_07293	502109076	multiple C2 and transmembrane domain-containing protein 2-like [Cicer arietinum]	85607	9.26	42	5	739	_	others	Yes	protein binding	+
Ca_07362	502109744	HUA2-like protein 3-like [Cicer arietinum]	146493	6.02	31	2	1321	_	others	Yes		
Ca_00840	502112102	2-Cys peroxiredoxin BAS1-like, chloroplastic-like isoform X2 [Cicer arietinum]	22641	4.95	120	3	201	_	others	No	catalytic activity	
Ca_00971	502112597	probable exocyst complex component 6-like [Cicer arietinum]	91308	5.86	81	4	805	_	others	No	cytoplasm	

Ca_00951	502112527	c-1-tetrahydrofolate synthase, cytoplasmic-like [Cicer arietinum]	25320	8.43	59	2	237	_	others	No	catalytic activity	
Ca_01002	502112711	protein PROLIFERA-like isoform X1 [Cicer arietinum]	71355	5.64	52	4	634	_	others	No	nucleotide binding	
Ca_00161	502077303	dynamin-related protein 3A-like [Cicer arietinum]	92310	6.05	49	3	835	_	_	No	nucleotide binding	
Ca_01177	502113385	signal peptidase complex subunit 3B-like [Cicer arietinum]	19143	9.01	36	2	167	YES(1-26)	S	Yes	protein metabolic process	
Ca_01240	502114478	tankyrase-2-like [Cicer arietinum]	43059	4.8	104	3	403	_	S	No	protein binding	
Ca_13636	502149153	dolichyl-diphosphooligosaccharide--protein glycosyltransferase 48 kDa subunit-like [Cicer arietinum]	47637	5.65	139	4	425	YES(1-19)	S	Yes	carbohydrate metabolic process	+
Ca_01237	502113611	hevein-like preproprotein-like [Cicer arietinum]	23254	5.65	115	2	208	YES(1-24)	S	Yes	response to biotic stimulus	
Ca_09977	502153472	peptidyl-prolyl cis-trans isomerase Pin1-like [Cicer arietinum]	13466	9.13	115	5	124	_	others	Yes	catalytic activity	
Ca_03646	502117049	single-stranded DNA-binding protein, mitochondrial-like [Cicer arietinum]	23656	8.81	103	3	212	_	_	No	DNA binding	+
Ca_08502	502118489	serine carboxypeptidase-like 27-like isoform X1 [Cicer arietinum]	50182	6.37	157	4	436	_	others	Yes	protein metabolic process	
Ca_26434	502146307	ran-binding protein 10-like [Cicer arietinum]	53205	5.15	43	2	468	_	others	No	protein binding	
Ca_12997	502163756	serine/threonine-protein phosphatase 2A 65 kDa regulatory subunit A beta isoform-like [Cicer arietinum]	65848	4.87	263	7	587	_	others	No	binding	
Ca_00286	502077826	stem-specific protein TSJT1-like [Cicer arietinum]	27854	6.3	159	3	251	_	others	No		
Ca_05433	502121015	diphosphomevalonate decarboxylase-like [Cicer arietinum]	46961	6.22	100	4	421	_	others	No	nucleotide binding	
Ca_21615	502121553	leukotriene A-4 hydrolase-like [Cicer arietinum]	69108	5.27	214	6	609	_	others	No	protein metabolic process	
Ca_15742	20975622	Putative ripening related protein	17581	5.96	59	3	152	_	others	Yes	response to biotic stimulus	
Ca_18651	502121454	chalcone--flavonone isomerase 1-like [Cicer arietinum]	24386	5.56	581	15	224	_	others	No	catalytic activity	
Ca_20739	657392498	actin-binding FH2 (formin-like) protein	204267	5.72	92	2	1870	_	_	Yes	nucleic acid binding	+
Ca_11396	502135172	adenosylhomocysteinase-like [Cicer arietinum]	53911	5.57	205	7	485	_	others	No	metabolic process	+
Ca_05761	502139241	aminopeptidase N-like [Cicer arietinum]	110069	5.91	383	11	973	_	_	No	protein metabolic process	+
Ca_09569	502140127	annexin D2-like [Cicer arietinum]	35494	5.21	365	13	315	_	others	No	lipid binding	+
Ca_06374	502142821	apoptosis inhibitor 5-like isoform X1 [Cicer arietinum]	63324	9.14	243	7	571	_	others	No	binding	
Ca_14788	502084904	beta-D-xylosidase 1-like [Cicer arietinum]	83203	8.23	422	10	758	_	others	No	carbohydrate metabolic process	+
Ca_10072	502156268	beta-fructofuranosidase	65097	8.31	118	3	573	YES(1-22)	S	Yes	carbohydrate metabolic process	
Ca_09535	502104741	bifunctional dihydroflavonol 4-reductase/flavanone 4-reductase-like [Cicer arietinum]	56472	5.43	74	2	503	_	others	No	catalytic activity	
Ca_05874	502138858	biotin carboxylase 1	59867	7.59	350	10	541	_	_	No	nucleotide binding	
Ca_04835	502131106	catalase-4-like isoform X2 [Cicer arietinum]	57097	6.97	386	13	492	_	others	No	catalytic activity	
Ca_18874	502149433	coatomer subunit beta-1-like [Cicer arietinum]	106779	5.79	537	13	950	_	others	No	cytoplasm	
Ca_15247	502098814	conserved oligomeric Golgi complex subunit 5-like [Cicer arietinum]	91320	7.1	86	4	830	_	_	No	cytoplasm	+
Ca_18347	502125689	cysteine proteinase RD21a-like [Cicer arietinum]	55944	5.69	89	2	495	YES(1-26)	S	Yes	protein metabolic process	+
Ca_03177	502151023	DEAD-box ATP-dependent RNA helicase 30-like [Cicer arietinum]	46833	5.23	469	12	413	_	others	No	nucleic acid binding	

Ca_04759	502130823	dehydrin ERD14-like [Cicer arietinum]	25527	5.21	82	4	225	_	others	Yes	response to abiotic stimulus	
Ca_15342	502152110	delta-1-pyrroline-5-carboxylate dehydrogenase 12A1	61850	6.17	280	10	553	_	_	No	catalytic activity	
Ca_10018	502156398	developmentally regulated G-protein 2-like [Cicer arietinum]	44974	9.01	61	3	399	_	others	No	nucleotide binding	
Ca_03055	502150750	dihydrolipoyl dehydrogenase	53767	6.69	875	18	503	_	_	No	nucleotide binding	+
Ca_00610	502079063	dihydroxy-acid dehydratase-like [Cicer arietinum]	64925	6	119	5	601	_	_	No	catalytic activity	+
Ca_03978	502136321	dipeptidyl peptidase 8-like [Cicer arietinum]	87563	6.41	65	2	771	_	others	No	protein metabolic process	+
Ca_05156	502141886	DNA replication licensing factor MCM3 homolog [Cicer arietinum]	87847	6.21	93	3	786	_	others	No	nucleotide binding	
Ca_04809	502130990	dolichyl-diphosphooligosaccharide--protein glycosyltransferase subunit 1A-like [Cicer arietinum]	69135	7.73	115	3	615	YES(1-24)	S	No	carbohydrate metabolic process	+
Ca_05293	657389535	dynamin 3A-like protein [Medicago truncatula]	85068	6.16	53	3	770	_	_	No	nucleotide binding	
Ca_21231	502127455	endoglucanase 9-like [Cicer arietinum]	55021	8.76	98	3	493	YES(1-23)	S	Yes	carbohydrate metabolic process	
Ca_01630	502132673	far upstream element-binding protein 2-like [Cicer arietinum]	61484	5.42	27	1	590	_	others	Yes	RNA binding	
Ca_06452	502143107	ferredoxin--nitrite reductase	57486	6.45	238	8	507	_	others	No	catalytic activity	
Ca_03035	502150796	galactokinase-like [Cicer arietinum]	53222	5.57	321	10	479	_	others	No	nucleotide binding	
Ca_04783	692112456	glucanase [Cicer arietinum]	49069	4.96	60	3	454	YES(1-21)	S	Yes	carbohydrate metabolic process	+
Ca_05751	502139270	GMP synthase [glutamine-hydrolyzing]-like [Cicer arietinum]	59888	6.15	190	3	536	_	others	No	nucleotide binding	+
Ca_16834	502163100	helicase SRCAP-like [Cicer arietinum]	44447	11.01	59	3	422	_	_	Yes	DNA binding	
Ca_18681	604299836	hypothetical protein MIMGU_mgv1a015002mg [Erythranthe guttata]	50310	4.89	109	5	447	_	others	No	hydrolase activity	
Ca_02050	593787796	hypothetical protein PHAVU_002G029800g [Phaseolus vulgaris]	72901	6.08	293	8	656	_	_	No	nucleotide binding	
Ca_20058	502128833	imidazole glycerol phosphate synthase hisHF	62634	5.97	141	7	571	_	_	No	catalytic activity	+
Ca_04000	502136423	importin subunit beta-1-like [Cicer arietinum]	97151	4.66	53	3	869	_	others	No	protein binding	
Ca_02190	357482733	Indole-3-acetic acid-amido synthetase GH3.3 [Medicago truncatula]	63612	5.91	391	11	558	_	others	No	biological_process	
Ca_04942	502131502	isoflavone reductase-like protein-like isoform X2 [Cicer arietinum]	34214	5.85	406	11	310	_	others	No		
Ca_15413	502149347	kinesin-related protein 4-like isoform X1 [Cicer arietinum]	120469	5.73	34	3	1081	_	_	Yes	nucleotide binding	
Ca_05855	502138932	luminal-binding protein-like [Cicer arietinum]	73772	5.02	815	19	667	YES(1-27)	S	No		+
Ca_07568	502134482	lysosomal beta glucosidase-like [Cicer arietinum]	77025	8.81	111	2	699	YES(1-23)	S	No	carbohydrate metabolic process	+
Ca_06305	502142623	monothiol glutaredoxin-S17-like [Cicer arietinum]	54226	5.28	117	3	490	_	others	No	cellular homeostasis	
Ca_21687	502087362	NEDD8 ultimate buster 1-like [Cicer arietinum]	61614	5.24	89	1	551	_	others	No	protein binding	
Ca_15163	502145834	nicalin-1-like [Cicer arietinum]	62614	5.85	120	5	564	_	S	No	protein metabolic process	
Ca_27651	502135559	nitronate monooxygenase-like [Cicer arietinum]	35556	5.76	205	5	330	_	others	No	catalytic activity	
Ca_02103	502161028	nuclear autoantigenic sperm protein-like isoform X2 [Cicer arietinum]	41562	4.48	121	3	388	_	others	Yes	protein binding	
Ca_11084	502144698	nucleolin 2-like isoform X1 [Cicer arietinum]	61435	5.13	93	4	576	_	others	Yes	nucleic acid binding	

Ca_18681	502126785	nudix hydrolase 3-like [Cicer arietinum]	50310	4.89	109	5	447	_	others	No	nucleotide binding	
Ca_08002	502080118	obg-like ATPase 1-like [Cicer arietinum]	46960	6.58	126	6	415	_	others	No	protein metabolic process	+
Ca_11539	502162590	oligopeptidase A-like [Cicer arietinum]	79273	5.3	525	14	700	_	others	No	metabolic process	+
Ca_01657	502132598	omega-amidase NIT2-like isoform X2 [Cicer arietinum]	33042	5.76	284	6	302	_	_	No	protein binding	
Ca_11630	502094215	pentatricopeptide repeat-containing protein At1g26460	68468	6.69	83	4	609	_	_	No	protein metabolic process	
Ca_11724	502155914	peptidyl-prolyl cis-trans isomerase FKBP62-like [Cicer arietinum]	64553	5.26	300	10	577	_	others	Yes	biological_process	
Ca_14514	502154817	pescadillo homolog isoform X2 [Cicer arietinum]	69714	8.96	124	5	604	_	others	No	protein binding	
Ca_02881	502080754	phospholipase D alpha 1-like isoform X1 [Cicer arietinum]	92595	5.48	81	4	809	_	others	No	nucleotide binding	
Ca_06476	502143621	polyadenylate-binding protein 2-like [Cicer arietinum]	71610	8.37	179	5	659	_	_	Yes	protein binding	
Ca_07291	571557239	polygalacturonase inhibitor-like [Glycine max]	36683	6.64	71	2	329	YES(1-20)	S	Yes	catalytic activity	+
Ca_06788	502151252	primary amine oxidase-like [Cicer arietinum]	57638	6.16	128	5	508	_	others	Yes	hydrolase activity	+
Ca_20994	525314360	probable inactive purple acid phosphatase 29-like [Cicer arietinum]	47774	5.89	182	5	423	YES(1-15)	others	Yes	cellular homeostasis	
Ca_26012	502155094	probable nucleoredoxin 1-like [Cicer arietinum]	60368	4.82	133	5	529	_	others	No		
Ca_07421	502133642	probable pectate lyase 5-like [Cicer arietinum]	45398	6.66	237	6	407	YES(1-24)	S	Yes	protein metabolic process	+
Ca_09985	502153431	probable serine protease EDA2-like [Cicer arietinum]	57092	5.13	80	2	501	YES(1-19)	S	No	hydrolase activity	
Ca_02072	502161070	probable Xaa-Pro aminopeptidase P-like [Cicer arietinum]	73669	5.75	30	1	655	_	others	No	DNA binding	+
Ca_15583	502129176	proliferating cell nuclear antigen-like [Cicer arietinum]	29828	4.69	188	7	266	_	others	No	protein metabolic process	
Ca_12860	502089910	proliferation-associated protein 2G4-like isoform X2 [Cicer arietinum]	43832	6.12	264	11	394	_	others	No	nucleotide binding	
Ca_10083	502156238	protein ROOT HAIR DEFECTIVE 3 homolog 1-like [Cicer arietinum]	90785	5.78	102	4	812	_	others	No	cytoplasm	
Ca_11377	502135229	protein transport protein SEC23-like isoform X1 [Cicer arietinum]	89523	5.98	111	3	800	_	others	No	protein metabolic process	
Ca_01648	502132618	puromycin-sensitive aminopeptidase-like [Cicer arietinum]	99216	5.34	122	5	875	_	others	No	response to biotic stimulus	+
Ca_14775	6469115	putative ABA-responsive protein [Cicer arietinum]	16660	5.17	103	5	157	_	others	Yes	response to biotic stimulus	+
Ca_15741	48714607	putative Bet v I family protein, partial [Cicer arietinum]	17736	5.28	165	4	152	_	others	No	protein binding	
Ca_11389	502135187	selenium-binding protein 1-like [Cicer arietinum]	54623	5.34	192	5	488	_	others	Yes	RNA binding	
Ca_05679	502139530	signal recognition particle 68 kDa protein-like isoform X1 [Cicer arietinum]	68719	8.78	192	5	603	_	others	No	catalytic activity	
Ca_22314	502093007	spermidine synthase 1-like [Cicer arietinum]	37587	5.08	125	5	340	_	others	No	protein metabolic process	
Ca_11029	502144897	subtilisin-like protease-like [Cicer arietinum]	83237	9.02	176	5	776	YES	S	Yes	nucleotide binding	+
Ca_20260	502158017	SUMO-activating enzyme subunit 2-like [Cicer arietinum]	70113	5.36	48	3	628	_	S	No	protein binding	
Ca_18324	502086242	translocation protein SEC63 homolog [Cicer arietinum]	69847	5.39	39	2	618	_	S	No	protein binding	
Ca_05400	502121130	transportin-1-like [Cicer arietinum]	101042	4.92	87	2	893	_	others	No	cell	+
Ca_01949	502161411	vacuolar protein sorting-associated protein 35A-like [Cicer arietinum]	93754	5.23	44	2	824	_	others	No	nucleotide binding	
Ca_06306	502142629	vesicle-fusing ATPase-like isoform X2 [Cicer arietinum]	105083	5.7	502	17	936	_	others	No	protein binding	+
Ca_20629	502085897	WD-40 repeat-containing protein MSI4-like [Cicer arietinum]	56601	6.19	55	3	504	_	others	Yes	carbohydrate metabolic process	

Ca_04994	502131707	xylulose kinase-like [Cicer arietinum]	62102	5.87	127	4	559	_	others	No	nucleotide binding	
Ca_01103	502113098	GTP-binding protein SAR1A-like [Cicer arietinum]	22070	6.43	146	5	193	_	S	No	protein metabolic process	+
Ca_07155	502108069	10 kDa chaperonin-like [Cicer arietinum]	9543	9.65	66	3	89	_	_	No	protein metabolic process	+
Ca_01076	502112993	aspartic proteinase-like isoform X1 [Cicer arietinum]	56224	5.91	138	5	508	_	S	Yes		
Ca_01103	502113098	GTP-binding protein SAR1A-like [Cicer arietinum]	22070	6.43	146	5	193	_	S	No	catalytic activity	+
Ca_07830	502115428	RAN GTPase-activating protein 1-like [Cicer arietinum]	58095	4.58	397	12	530	_	_	No	metabolic process	
Ca_11870	502159003	cysteine synthase-like isoform X1 [Cicer arietinum]	34369	5.92	251	8	325	_	others	No	lipid binding	+
Ca_00455	502078511	serine hydroxymethyltransferase	56940	6.65	295	7	520	_	_	No	carbohydrate metabolic process	
Ca_19807	671775276	protease inhibitor/seed storage/ltp family [Cicer arietinum]	13812	9.02	30	1	130	Yes(1-23)	S	Yes	generation of precursor metabolites and energy	
Ca_00767	356505332	fructose-bisphosphate aldolase, cytoplasmic isozyme [Glycine max]	37151	5.81	52	1	343	_	others	No	generation of precursor metabolites and energy	+
Ca_12888	357452217	Phosphoenolpyruvate-carboxylase [Medicago truncatula]	106229	5.8	57	2	925	_	others	No	protein binding	
Ca_02491	357453687	Phosphoenolpyruvate carboxylase [Medicago truncatula]	107336	5.95	36	1	935	_	others	Yes	biosynthetic process	
Ca_11969	357510435	Eukaryotic translation initiation factor 3 subunit A [Medicago truncatula]	116792	8.95	45	2	992	_	others	Yes	protein binding	+
Ca_00006	502076624	methionine S-methyltransferase-like isoform X4 [Cicer arietinum]	145146	5.41	44	4	1296	_	others	Yes	structural molecule activity	+
Ca_00028	502076739	eukaryotic translation initiation factor 3 subunit C-like [Cicer arietinum]	105664	5.51	195	7	933	_	others	No	catalytic activity	+
Ca_00251	502077695	60S ribosomal protein L26-1-like [Cicer arietinum]	16699	11.02	39	1	146	_	_	No	nucleotide binding	
Ca_00642	502079142	putative lactoylglutathione lyase-like isoform X2 [Cicer arietinum]	32285	5.58	55	4	287	_	others	No	nucleotide binding	
Ca_02560	502082135	cell division cycle protein 48 homolog [Cicer arietinum]	90635	5.16	395	12	808	_	others	No	protein binding	+
Ca_02508	502082360	carbamoyl-phosphate synthase large chain-like [Cicer arietinum]	127363	5.23	47	4	1165	_	_	Yes	metabolic process	
Ca_02487	502082416	14-3-3-like protein B-like isoform X1 [Cicer arietinum]	31392	4.79	107	4	277	_	others	No	nucleotide binding	+
Ca_07124	502083283	aconitate hydratase 2, mitochondrial-like [Cicer arietinum]	101923	7.88	352	11	932	_	_	No		+
Ca_23118	502088040	leucine-rich repeat receptor-like serine/threonine-protein kinase At1g17230-like isoform X1 [Cicer arietinum]	105028	5.79	39	2	939	_	others	No		+
Ca_12633	502090294	actin-like [Cicer arietinum]	41906	5.31	87	5	377	_	others	Yes	catalytic activity	+
Ca_14693	502091451	probable pectate lyase 5-like [Cicer arietinum]	45598	6.93	44	2	405	Yes+(1-23)	S	No	protein binding	+
Ca_19158	502092083	probable glutamate dehydrogenase 3-like [Cicer arietinum]	46091	6.28	120	5	420	_	_	No	nucleotide binding	+
Ca_14960	502095463	E3 ubiquitin-protein ligase UPL1-like isoform X1 [Cicer arietinum]	397709	5.11	33	2	3594	_	_	No	catalytic activity	
Ca_14309	502096116	ubiquitin-activating enzyme E1 1-like isoform X2 [Cicer arietinum]	108763	5.32	116	3	973	_	others	No	biological_process	+
Ca_15641	502096332	peroxidase 4-like [Cicer arietinum]	37158	9.39	35	3	335	Yes+(1-37)	S	No	cytoskeleton	+
Ca_12497	502097328	60S acidic ribosomal protein P0-like [Cicer arietinum]	34312	5.21	45	3	321	_	others	No		
Ca_22693	502102666	actin-11-like [Cicer arietinum]	41974	5.23	103	5	377	_	others	No	molecular_function	+
Ca_20417	502104783	fasciclin-like arabinogalactan protein 10-like [Cicer arietinum]	43849	5.88	38	1	423	Yes+(1-23)	S	No	protein binding	
Ca_06139	502106723	vicilin-like [Cicer arietinum]	52124	5.62	140	5	458	Yes+(1-23)	S	No	nucleotide binding	+

Ca_07290	502109061	polygalacturonase inhibitor-like [Cicer arietinum]	38153	8.44	50	1	335	Yes+(1-22)	S	No	nucleotide binding	+
Ca_07352	502109411	LOW QUALITY PROTEIN: elongation factor Tu, mitochondrial-like [Cicer arietinum]	49174	6.58	69	2	450	_	_	No	carbohydrate binding	+
Ca_12054	502111218	cell division control protein 48 homolog D-like isoform X2 [Cicer arietinum]	90357	5.11	373	11	807	_	others	No	catalytic activity	+
Ca_01015	502112755	nodal modulator 1-like [Cicer arietinum]	130098	5.59	99	5	1196	Yes+(1-23)	S	No	binding	+
Ca_01173	502113361	glucose and ribitol dehydrogenase homolog 1-like [Cicer arietinum]	32289	6.6	36	1	293	_	others	No	signal transduction	+
Ca_01274	502113779	LOW QUALITY PROTEIN: translational activator GCN1-like [Cicer arietinum]	289142	6.6	38	3	2639	_	_	No	protein binding	
Ca_07732	502115754	TBC1 domain family member 15-like [Cicer arietinum]	48362	8.66	46	2	424	_	others	No	nucleotide binding	
Ca_08385	502118198	coatomer subunit alpha-1-like [Cicer arietinum]	133832	6.43	207	8	1193	_	_	No	nucleotide binding	
Ca_04392	502119038	LOW QUALITY PROTEIN: eukaryotic translation initiation factor 5B-like [Cicer arietinum]	147111	5.31	36	2	1342	_	others	No	nucleotide binding	+
Ca_04505	502119424	valine--tRNA ligase-like [Cicer arietinum]	117960	5.81	126	6	1028	_	others	No	nucleic acid binding	+
Ca_05504	502120770	glutamine--tRNA ligase-like [Cicer arietinum]	90748	6.06	76	4	794	_	others	No	nucleotide binding	+
Ca_18640	502121426	splicing factor 3B subunit 3-like [Cicer arietinum]	138504	5.35	272	7	1250	_	others	No	structural molecule activity	
Ca_20123	502122167	monodehydroascorbate reductase-like [Cicer arietinum]	47198	5.47	64	4	433	_	others	No	nucleus	+
Ca_20452	502122561	60S ribosomal protein L4-like [Cicer arietinum]	44763	10.43	162	8	406	_	others	No	metabolic process	
Ca_20011	502123419	intron-binding protein aquarius-like [Cicer arietinum]	183090	5.5	53	3	1587	_	others	No	catalytic activity	
Ca_09074	502125049	aspartate aminotransferase P2, mitochondrial-like [Cicer arietinum]	51223	8.55	30	2	467	_	_	Yes	protein binding	+
Ca_09169	502125338	glycine dehydrogenase [decarboxylating], mitochondrial-like isoform X1 [Cicer arietinum]	119268	8.25	99	4	1086	_	_	No	metabolic process	+
Ca_13404	502130011	clathrin heavy chain 1-like [Cicer arietinum]	194326	5.33	850	23	1703	_	others	No	biological_process	+
Ca_01885	502131988	aspartate aminotransferase 1-like [Cicer arietinum]	52506	7.25	198	6	478	_	_	Yes	catalytic activity	+
Ca_01813	502132222	60S acidic ribosomal protein P0-like [Cicer arietinum]	34145	5.42	106	3	322	_	others	No	metabolic process	
Ca_03927	502136156	alcohol dehydrogenase 1-like [Cicer arietinum]	41784	5.92	160	9	380	_	others	Yes	protein metabolic process	
Ca_04273	502137281	staphylococcal nuclease domain-containing protein 1-like isoform X1 [Cicer arietinum]	108608	6.83	314	10	989	_	_	No		
Ca_10391	502138201	26S proteasome non-ATPase regulatory subunit 1-like [Cicer arietinum]	109615	5.18	360	12	1007	_	S	No	protein metabolic process	
Ca_10428	502138331	cysteine-rich repeat secretory protein 12-like [Cicer arietinum]	26412	5.4	68	2	241	_	_	No	catalytic activity	+
Ca_05912	502138725	mitochondrial-processing peptidase subunit alpha-like [Cicer arietinum]	55147	5.81	39	2	510	_	_	Yes	generation of precursor metabolites and energy	+
Ca_05862	502138893	probable phosphoribosylformylglycinamidine synthase, chloroplastic/mitochondrial-like [Cicer arietinum]	154235	5.29	150	7	1407	_	_	No	membrane	
Ca_19873	502143864	phosphoenolpyruvate carboxylase-like [Cicer arietinum]	109595	5.46	52	3	958	_	others	No	nucleotide binding	
Ca_11237	502144106	prohibitin-1, mitochondrial-like [Cicer arietinum]	32482	9.47	56	2	294	_	others	Yes		+
Ca_11150	502144299	isoleucine--tRNA ligase, cytoplasmic-like [Cicer arietinum]	134338	5.96	152	8	1182	_	others	No	nucleotide binding	
Ca_11131	502144361	putative leucine-rich repeat-containing protein DDB_G0290503-like isoform X1 [Cicer arietinum]	153138	4.69	286	11	1356	_	others	No	protein binding	+
Ca_13881	502147205	leucine--tRNA ligase, cytoplasmic-like isoform X2 [Cicer arietinum]	123948	5.95	574	16	1090	_	others	Yes	protein binding	+

Ca_13488	502147633	heat shock 70 kDa protein 15-like [Cicer arietinum]	94672	5.22	645	15	851	_	others	No	protein binding	+
Ca_13490	502147635	heat shock 70 kDa protein 15-like [Cicer arietinum]	88414	5.12	491	13	793	_	others	No	protein binding	+
Ca_13486	502147645	coatomer subunit beta'-2-like [Cicer arietinum]	106029	4.84	51	4	932	_	_	No	nucleotide binding	
Ca_15410	502149340	coatomer subunit alpha-1-like isoform X1 [Cicer arietinum]	136977	6.55	293	12	1222	_	_	Yes	protein binding	
Ca_06746	502151359	116 kDa U5 small nuclear ribonucleoprotein component-like [Cicer arietinum]	110945	4.99	71	5	990	_	others	No	metabolic process	
Ca_17637	502156832	exportin-2-like [Cicer arietinum]	109731	5.18	113	3	970	_	_	No	nucleotide binding	+
Ca_01932	502161475	aconitate hydratase 1-like [Cicer arietinum]	99144	5.75	382	11	901	_	others	Yes	structural molecule activity	
Ca_10700	502161816	thioredoxin reductase 2-like [Cicer arietinum]	35194	5.91	49	1	325	_	others	No	nucleotide binding	+
Ca_10689	502161844	60S ribosomal protein L4-like [Cicer arietinum]	44940	10.4	153	6	407	_	others	Yes	nucleotide binding	
Ca_16809	502163295	uncharacterized protein LOC101509479 [Cicer arietinum]	139079	5.55	109	5	1292	_	others	No	structural molecule activity	
Ca_13004	502163739	DNA primase/helicase-like [Cicer arietinum]	76629	8.36	37	2	673	_	_	No	protein binding	
Ca_02175	525313620	40S ribosomal protein SA [Cicer arietinum]	33729	5	88	3	304	_	others	No	protein binding	+
Ca_12469	565380123	clathrin heavy chain 1-like [Solanum tuberosum]	189058	5.23	232	11	1662	_	others	Yes	generation of precursor metabolites and energy	+
Ca_09027	593692484	hypothetical protein PHAVU_006G069700g [Phaseolus vulgaris]	166353	5.21	179	8	1463	_	_	No	carbohydrate metabolic process	
Ca_19382	657376939	E1 subunit-like 2-oxoglutarate dehydrogenase [Medicago truncatula]	128395	6.7	546	15	1131	_	_	No	nucleotide binding	+
Ca_05018	671775248	chitinase [Cicer arietinum]	39432	7.72	115	3	364	Yes(1-24)	S	No		+
Ca_12469	565380123	clathrin heavy chain 1-like [Solanum tuberosum]	189058	5.23	232	11	1662	_	others	Yes		+
Ca_11078	727580998	alanine--tRNA ligase-like [Camelina sativa]	112391	5.85	108	3	1012	_	others	No		+

[a] Accession no. as in MSDB database. [b] S in the column indicates the proteins predicted in secretory fraction. [c] GO program used for the prediction of function of protein ontologically. [d] '+' in the column indicates the presence of protein in previous studies in secretory fraction.

Supplementary Table 4. List of primers

Primers	Forward	Reverse
CaRRP1pGEM	5’-CGCGGATCCATGGTTCTAGCTGGTAAACT-3’	5’-ATAAGAATGCGGCCGCCTATGCCTTGACAAGATTA-3′
CaRRP1pYES2	5’-CGCGGATCCATGGTTCTAGCTGGTAAACT-3’	5’-ATAAGAATGCGGCCGCCTATGCCTTGACAAGATTA-3′
CaRRP1RT	5′- CACCTGCTTCCAAGTTTTACAATCT -3′	5′- TCTTTCACAATGGTTTTGCACTTC -3′
CaEF1	5′- TCCACCACTTGGTCGTTTTG -3′	5′- CTTAATGACACCGACAGC -3′
CaRRP1pENTR	5’-CACCATGGTTCTAGCTGGTAAACTTA-3’	5’-TGCCTTGACAAGATTACCATCCAAGT-3′
